# RACK1 mediates rewiring of intracellular networks induced by hepatitis C virus infection

**DOI:** 10.1371/journal.ppat.1008021

**Published:** 2019-09-16

**Authors:** Jae Seung Lee, Keisuke Tabata, Woan-Ing Twu, Md Shafiqur Rahman, Hee Sun Kim, Jin Bae Yu, Min Hyeok Jee, Ralf Bartenschlager, Sung Key Jang

**Affiliations:** 1 Division of Integrative Bioscience & Biotechnology, POSTECH Biotech Center, POSTECH, Nam-gu, Pohang-si, Gyeongsangbuk-do, Rep. of KOREA; 2 Department of Infectious Diseases, Molecular Virology, Heidelberg University, Heidelberg, Germany; 3 Department of Life Sciences, POSTECH Biotech Center, POSTECH, Nam-gu, Pohang-si, Gyeongsangbuk-do, Rep. of KOREA; 4 Division Virus-Associated Carcinogenesis, German Cancer Research Center, Heidelberg, Germany; The University of Chicago, UNITED STATES

## Abstract

Hepatitis C virus (HCV) is a positive-strand RNA virus replicating in a membranous replication organelle composed primarily of double-membrane vesicles (DMVs) having morphological resemblance to autophagosomes. To define the mechanism of DMV formation and the possible link to autophagy, we conducted a yeast two-hybrid screening revealing 32 cellular proteins potentially interacting with HCV proteins. Among these was the Receptor for Activated Protein C Kinase 1 (RACK1), a scaffolding protein involved in many cellular processes, including autophagy. Depletion of RACK1 strongly inhibits HCV RNA replication without affecting HCV internal ribosome entry site (IRES) activity. RACK1 is required for the rewiring of subcellular membranous structures and for the induction of autophagy. RACK1 binds to HCV nonstructural protein 5A (NS5A), which induces DMV formation. NS5A interacts with ATG14L in a RACK1 dependent manner, and with the ATG14L-Beclin1-Vps34-Vps15 complex that is required for autophagosome formation. Both RACK1 and ATG14L are required for HCV DMV formation and viral RNA replication. These results indicate that NS5A participates in the formation of the HCV replication organelle through interactions with RACK1 and ATG14L.

## Author summary

All positive-strand RNA viruses replicate their genomes in distinct membrane-associated compartments designated replication organelles. The compartmentalization of viral replication machinery allows the enrichment and coordination of cellular and viral factors required for RNA replication and the evasion from innate host defense systems. Hepatitis C virus (HCV), a prototype member of the *Flaviviridae* family, rearranges intracellular membranes to construct replication organelles composed primarily of double-membrane vesicles (DMVs) which are morphologically similar to autophagosomes. Nonstructural protein 5A (NS5A), which is essential for HCV replication, induces DMV formation. Here, we report that NS5A triggers DMV formation through interactions with RACK1 and components of the vesicle nucleation complex acting at the early stage of autophagy. These results illustrate how a virus skews cellular machineries to utilize them for its replication by hijacking cellular proteins through protein-protein interactions. This research sheds light on the molecular basis of replication organelle formation by HCV and possibly other viruses employing organelles with DMV morphology.

## Introduction

Liver diseases caused by hepatitis C virus (HCV) constitute a significant health burden worldwide. Around 71 million people are chronically infected with HCV [1, 2], and more than 350,000 annual deaths are due to HCV infection, in most cases as a result of HCV-related liver cirrhosis and hepatocellular carcinoma (HCC). Although antiviral drugs allow HCV elimination in the vast majority of infected individuals, it is becoming increasingly clear that global eradication will require a vaccine that, however, is not yet available.

HCV is a *Hepacivirus* genus member, which belongs to the *Flaviviridae* family [3]. The HCV genome is a 9.6 kb long single-stranded RNA of positive sense encoding 10 or more viral proteins. The viral proteins are synthesized as a polyprotein via an internal ribosome entry site (IRES) residing in the 5′ untranslated region (5′UTR). The polyprotein is proteolytically cleaved into functional proteins by cellular and virus-encoded proteinases [4].

All known positive-strand RNA viruses to date replicate their genome in distinct membrane-associated compartments called replication organelles. This compartmentalization of replication machinery allows the enrichment and coordination of cellular and viral factors required for RNA replication, and the evasion from innate host defense systems recognizing non-self RNAs such as 5′-triphosphorylated RNAs and double-stranded RNAs [5, 6]. Two distinct morphotypes of replication organelles have been reported, corresponding to the ‘invaginated vesicles/spherule type’ and the ‘double membrane vesicle (DMV) type’ [7]. DMVs are the major component of the HCV replication organelle [8]. Among the HCV proteins, nonstructural protein 5A (NS5A), which is essential for HCV replication, was shown to induce DMV formation [9]. Consistent with this function, antiviral drugs targeting NS5A inhibit DMV formation [10]. Further investigation into the NS5A-mediated DMV formation revealed that NS5A domain 1 is required for the DMV formation [8]. Other viral protein(s) such as nonstructural protein 4B (NS4B) augments DMV formation activity of NS5A [7].

NS5A of HCV is a multi-functional protein that participates in viral RNA replication, virus assembly, and virion particle release, influencing several cellular processes such as apoptosis, stress-responses, immune responses, and the cell cycle [11]. NS5A is composed of an amino-terminal amphipathic α-helix tethering the protein to intracellular membranes, a highly structured domain 1 (D1), and two intrinsically unfolded domains (D2 and D3), separated from each other and D1 via low-complexity sequences (LCSs) [4]. Several lines of evidence, including the presence of active replicase complexes on DMV membranes, argue that DMVs are the HCV RNA replication site [9, 12]. However, the molecular basis of the NS5A-mediated DMV formation and HCV RNA replication remains to be elucidated.

Receptor for Activated C Kinase 1 (RACK1; also designated GNB2L1) is a member of the tryptophan-aspartate repeat (WD-repeat), family and shares significant homology to the β subunit of G-proteins. RACK1 plays essential roles in intracellular trafficking, anchoring proteins at particular locations, and stabilizing protein activity [13]. RACK1 is also a constituent of the eukaryotic ribosome. A cryo-electron microscopic study revealed that RACK1 is localized at the head region of the 40S ribosomal subunit in the vicinity of the mRNA exit channel [14]. Recently, RACK1 was reported to promote autophagy by enhancing the formation of ATG14L-Beclin1-Vps34-Vps15 complex under starvation conditions [15]. This complex herein referred to as “vesicle nucleation complex,” is a critical component of autophagy induction [16–18]. Genome-wide small interfering (si) RNA screening experiments identified RACK1 as host dependency factor for HCV RNA replication, but not RNA translation [19], but the underlying mechanism has not been addressed.

Autophagy is an evolutionary conserved catabolic mechanism for the degradation of long-lived organelles and cytoplasmic materials and is crucial for cell homeostasis. Considering the morphological similarities between HCV-induced DMV and autophagosome, it has been proposed that autophagy plays a role in the biogenesis of viral replication compartments [5]. Increasing evidence has shown that the activation of autophagy by HCV is required for HCV replication owing to its contribution to the establishment of replication organelles and modulation of innate immunity [20–25]. The increase of autophagy flux is triggered by either HCV NS4B or NS5A [26–30]. In contrast, DMV formation is induced by NS5A, although the induction efficiency is greatly improved by other HCV proteins [9].

Here we sought to identify cellular proteins participating in HCV replication by using the yeast two-hybrid screening method that is often employed to identify the protein(s) directly interacting with a protein of interest [31, 32]. We used HCV NS3, NS5A, and Core as baits and identified, amongst others, RACK1. RACK1 directly interacted with the NS5A domain 1 and showed the most potent effect on HCV replication. In order to understand how RACK1 participates in HCV RNA replication, we investigated the potential role of RACK1 in the autophagy induction by NS5A. We revealed that RACK is required for the NS5A-mediated autophagy induction. Both NS5A and RACK1 associate with the ATG14L-Beclin1-Vps34-Vps15 complex that is a crucial complex for autophagy induction. Interestingly, the interaction between NS5A and ATG14L was affected by RACK1. Depletion of either RACK1 or ATG14L decreases the number of DMVs generated by HCV non-structural proteins and dampens HCV RNA replication. Our results indicate that NS5A facilitates HCV RNA replication by triggering autophagy to construct RNA replication organelles through the interactions with RACK1 and the components of the vesicle nucleation complex.

## Results

### Identification of host proteins binding with HCV proteins

To investigate host factors involved in HCV life cycle, we performed yeast two-hybrid screening [32] using HCV core, NS3 and NS5A proteins as baits and human thymus and liver cDNA libraries as preys. Thirty-two host proteins were identified as potential human proteins interacting with HCV proteins (S1 Table). Based on their subcellular localizations and functions, 7 proteins were selected as potential candidates participating in HCV proliferation (Table 1).

**Table 1:** Representatives of host proteins potentially interacting with HCV proteins.

No	Gene Symbol	Description	Y2H^a^ binding partner	Uniprot
1	ANPEP	Alanyl (membrane) aminopeptidase	Core	P15144
2	AOX1	Aldehyde oxidase 1	NS3	Q06278
3	BAAT	Bile Acid-CoA: Amino Acid N-Acyltransferase	NS5A	Q14032
4	CTSB	Cathepsin B	NS5A	P07858
5	CYP2C8	Cytochrome P450 Family 2 Subfamily C Member 8	Core	P10632
6	ISOC2	Isochorismatase Domain Containing 2	NS5A	Q96AB3
7	RACK1	Receptor For Activated C Kinase 1	NS5A	P63244

List of proteins identified by yeast two-hybrid system as host proteins potentially interacting with Core, NS3, and NS5A.

^a^ Y2H: Yeast two-hybrid

**Fig 1:**
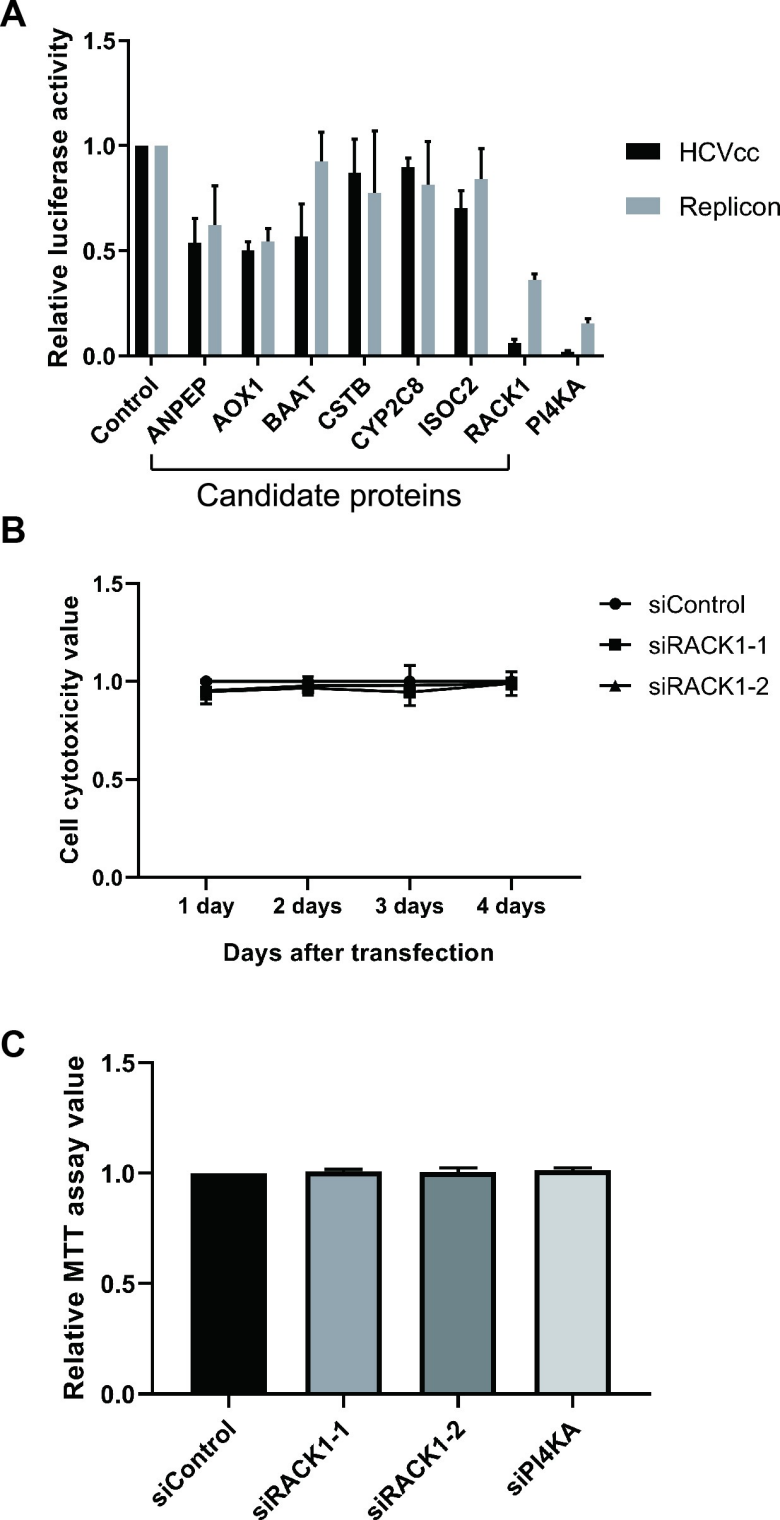
Effects of siRNAs on HCVcc and HCV replicon. (A) Effects of siRNAs targeting 7 host proteins on HCV proliferation. siRNAs were transfected into Huh7.5.1 cells or HCV 1b replicon-containing cells. JFH1-ad34-5A-Rluc (an HCVcc) was inoculated (0.1 MOI) 48 h after siRNA transfection, and further cultivated for 2 days. The cells were harvested and then lysed for luciferase assay. In the replicon system, the cells were lysed 72 h after siRNA transfection, and luciferase activities were measured. PI4KA was used as a positive control. Columns and bars represent the mean and standard deviation (SD) values of 3 independent experiments. (B, C) RACK1 knockdown does not affect cell proliferation. (B) Cytotoxic effect of RACK1 knockdown was monitored by the adenylate kinase (AK) detection method (ToxiLight, [63, 64]). Two siRNAs against RACK1 were transfected into Huh7.5.1 cells. The media were collected from cell cultures to measure AK activity at different time points after siRNA transfection (1 to 4 days). Three independent experiments were performed. (C) Cell viability test by MTT assay [65]. siRNAs targeting RACK1 or PI4KA were transfected into Huh7 cells containing an HCV 2a replicon. 3 days after siRNA transfections, the cells were cultivated with media containing methylthiazolyldiphenyl-tetrazolium bromide (MTT, 0.5 mg/ml) for 2 h, and the absorbance of media at 590 nm was measured. Three independent experiments were performed.

To validate the relevance of these host factors for HCV replication, we performed knockdown experiments with siRNAs targeting each of the 7 proteins. HCVcc and HCV replicon systems were used to monitor the participation of the proteins in HCV proliferation (Fig 1A). Among the host factors tested, knockdown of RACK1 showed the most potent inhibition of HCV proliferation in both experimental systems. However, RACK1 depletion did not affect cell proliferation (Fig 1B and 1C). Therefore, we conducted subsequent studies on RACK1 and its role in the HCV life cycle.

### RACK1 is required for HCV RNA replication

**Fig 2:**
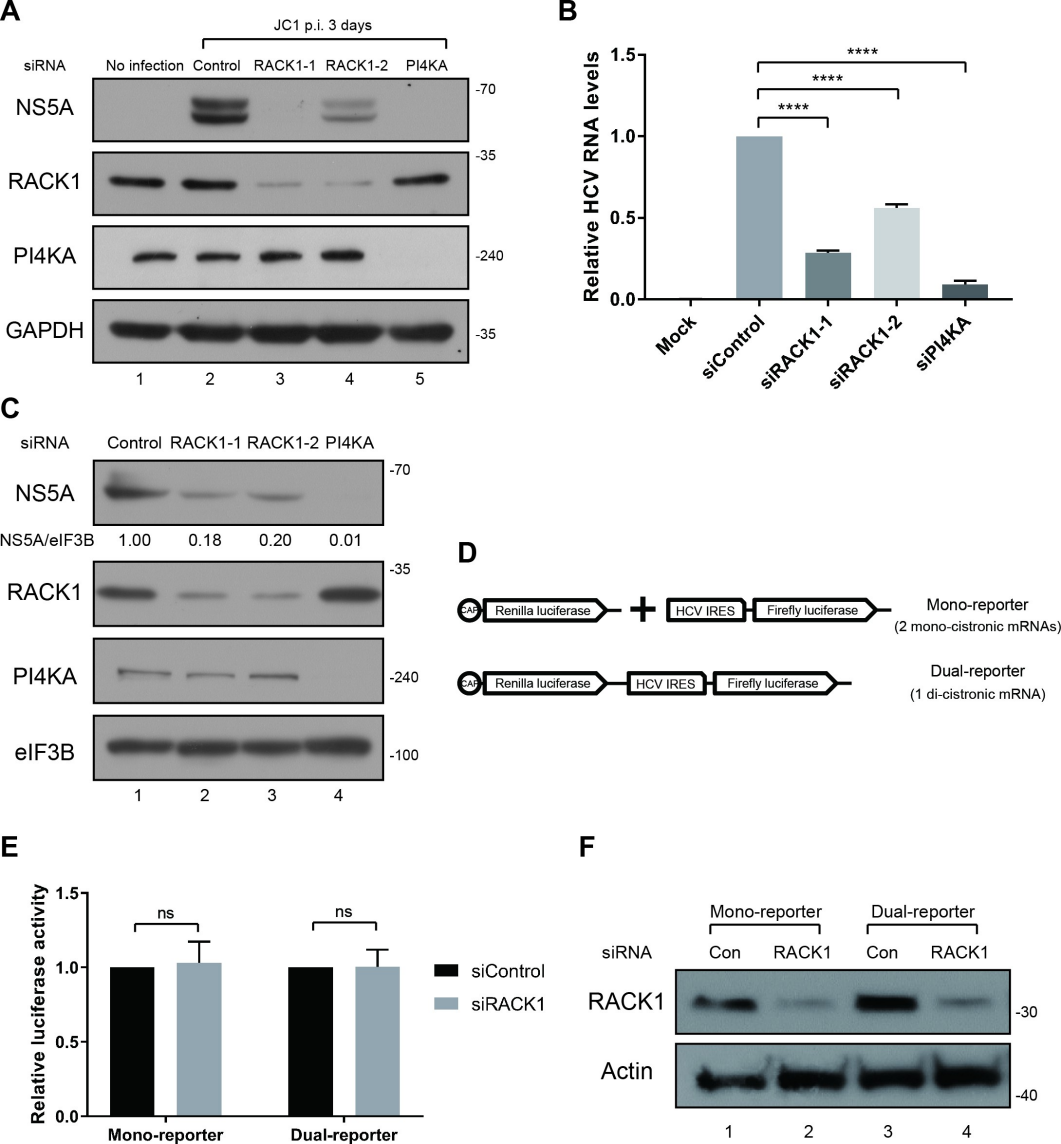
RACK1 is required for HCV replication. (A, B) siRNAs against RACK1 were transfected to Huh7.5.1 cells, and JC1 was inoculated (1 MOI) 48 h after transfection. Viral proliferation was monitored by Western blotting, and quantitative RT-PCR measured viral RNA levels 3 days after infection. Columns and bars represent the mean and SD values of 3 independent experiments. P value was less than 0.0001 by the analysis with ordinary one-way ANOVA multiple comparisons. (C) siRNAs against RACK1 and PI4KA were transfected to genotype 2a replicon cells. 72 hours later, viral protein levels and depleted protein levels were analyzed by Western blotting. The relative levels of NS5A, which were normalized by eIF3B, are given below each lane. The NS5A level in control siRNA transfected cells is set to 1. (D) Schematic diagrams of reporter mRNAs used in testing the translation function of HCV IRES. (E) Effects of RACK1 on HCV IRES-dependent translation. RNA reporters synthesized *in vitro* were transfected into RACK1 depleted Huh7 cells, and the luciferase activities were measured 4 h after transfection. HCV IRES-dependent translation value was normalized by cap-dependent translation value. Data represent the mean and SD of at least 3 independent experiments. ns stands for non-significant difference. (F) Knockdown efficiency of RACK1 siRNA was monitored by Western blotting in Huh7 cells transfected with reporter RNAs.

We confirmed the role of RACK1 in HCV life cycle by knockdown experiments with two different siRNAs against RACK1. In both cases, NS5A protein levels were reduced in Huh7.5.1 cells infected with HCVcc (JC1) 3 days post-infection (Fig 2A). Similarly, HCV RNA levels were reduced in the RACK1-depleted cells, and best detectable 3 days post-infection (Fig 2B). Consistent with the effects of siRNAs against RACK1 on HCVcc, RACK1 deletion dampened the replication of an HCV subgenomic replicon (genotype 2a) monitored by NS5A protein levels (Fig 2C).

We further validated the role of RACK1 in HCV replication through depletion and reconstitution experiments using Huh7 cell lines stably expressing either control GFP or GFP-fused RACK1 (GFP-RACK1) proteins (S1C and S1D Fig). The GFP and GFP-RACK1 genes do not encode the 3′ untranslated region (3′UTR) of endogenous RACK1 gene, wich contain the target sites for siRACK1-3 and siRNACK1-4. All siRNAs targeting the coding regions (siRACK1-1 and siRACK1-2) and the 3′UTR (siRACK1-3 and siRACK1-4) of endogenous RACK1 mRNAs reduced the levels of endogenous RACK1 protein (S1A Fig) and HCV RNA replication (S1B Fig). In rescue experiments, siRACK1-3 and siRACK1-4 reduced HCV replication in control cells expressing GFP, but not in cells expressing GFP-RACK1 of HCV (S1C and S1D Fig). This clearly demonstrates that RACK1 is required for HCV replication.

To determine whether HCV RNA translation is affected, we investigated the role of RACK1 in HCV IRES-dependent translation using mono- and di-cistronic mRNA reporter systems (Fig 2D). RACK1 knockdown did not affect HCV IRES-dependent translation (Fig 2E and 2F). Our results are consistent with those reported in a paper suggesting that HCV IRES-dependent translation is not affected by RACK1 [19]. We further investigated whether RACK1 affects the IRES-dependent translation in the presence of other viral proteins since HCV IRES activity was reported to be modulated by NS5A [33–35]. For this purpose, we used a HCV replicon RNA containing firefly luciferase gene and replication defective mutations within the active site of NS5B (ΔGDD). This replicon RNA was co-transfected with a capped reporter RNA containing *Renilla* luciferase gene as a reference mRNA. No significant change in translation of replicon RNA was detected in RACK1-depleted cells compared with RACK1-undepleted cells even at 4 and 6 h after RNA transfections when viral proteins were synthesized in the cells (S2 Fig). The results indicate that RACK1 does not affect HCV IRES-dependent translation even in the presence of viral proteins.

### RACK1 interacts with the domain 1 of HCV NS5A

**Fig 3:**
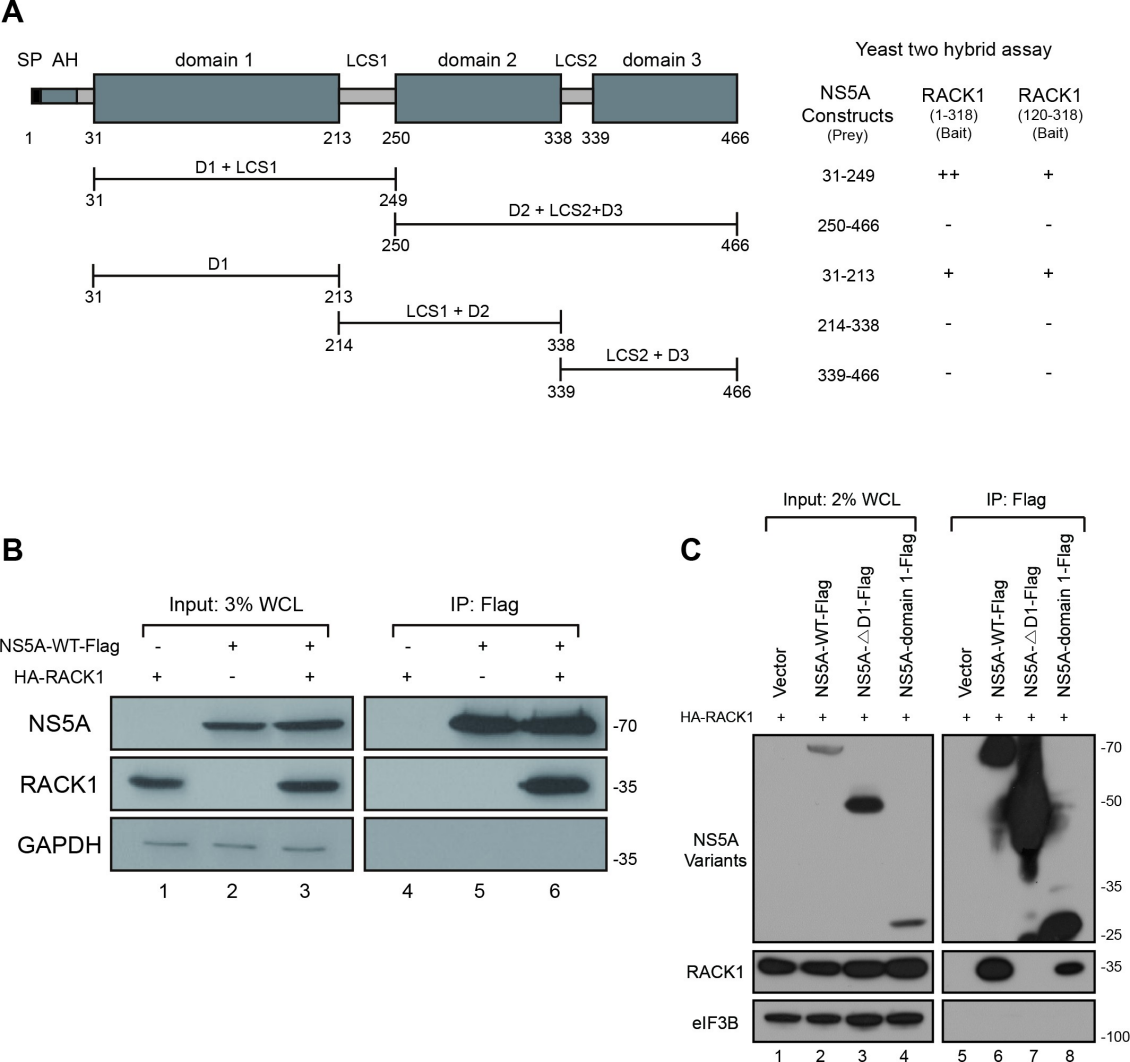
RACK1 interacts with the domain 1 of NS5A. (A) A schematic diagram of NS5A domain structure is shown on the top. SP, signal peptide; AH, amphipathic helix; LCS, low complexity sequence. The NS5A expression constructs used in the yeast two-hybrid assays to map the RACK1-binding site in NS5A are given below. Note that the AH was absent in all constructs to allow the nuclear localization of the prey proteins. The RACK1 full length (aa 1–318) and a RACK1 partial form (aa 120–318) were used as baits. ‘+’ indicates that a positive signal was observed from either *ADE2* or *URA3* reporter. ‘++’ indicates that positive signals were observed from both *ADE2* and *URA3* reporters (S1 Fig). (B) Co-immunoprecipitation of NS5A and RACK1. NS5A-WT-Flag and HA-RACK1 DNA were co-transfected in Huh7 cells. 48 hours post-transfection, the cells were lysed, and pulldown experiments were performed with a Flag-resin. The resin-bound proteins were analyzed by Western blotting. 3% of Flag-captured proteins were loaded onto the input lanes. At least 3 independent experiments were performed. WCL, whole cell lysate. (C) Plasmids encoding HA-tagged RACK1 and Flag-tagged NS5A variants were co-transfected into HEK293FT cells. The cells were harvested and lysed 48 h after transfection. Flag-tagged proteins were pulled down by a Flag-resin, and resin-bound proteins were analyzed by Western blotting. 2% of Flag-captured proteins were loaded onto the input lanes. 3 independent experiments were performed. WCL, whole cell lysate.

The region in HCV NS5A required for the interaction with RACK1 was determined by using the yeast two-hybrid system (Fig 3A). Various prey vectors encoding different parts of NS5A and two bait vectors expressing different parts of RACK1 were generated, and two-hybrid analyses were performed (Fig 3A and S3 Fig). The prey vectors expressing the domain 1 of NS5A (aa 31–213) and the bait vectors expressing the C-terminal part of RACK1 (aa 120–318) showed positive signals in the two-hybrid system (Fig 3A, right panel). The results indicate that domain 1 of NS5A and aa 120–318 of RACK1 participate in the protein-protein interaction between NS5A and RACK1.

We confirmed the role of NS5A domain 1 in the interaction with RACK1 by co-immunoprecipitation experiments using mammalian cells (Fig 3B and 3C). RACK1 protein was co-precipitated with the wild type (WT) (Fig 3B, lane 6) and the domain 1 of NS5A protein (Fig 3C, lanes 8). In contrast, RACK1 protein was not co-precipitated with an NS5A with a deletion of domain 1 (NS5A-ΔD1-Flag; lane 7 in Fig 3C) even though the expression level of this polypeptide was higher than other derivatives of NS5A (Fig 3B, compare lane 3 with lanes 2 and 4). The results indicate that domain 1 of NS5A is necessary and sufficient for the interaction with RACK1.

### NS5A induces autophagy through a RACK1 mediated pathway

In order to decipher the molecular basis of how NS5A and RACK1 contribute to HCV RNA replication, we focused on the autophagy pathway since autophagy-related proteins are required for HCV replication [21, 24]. It is also known that DMVs induced by HCV are structurally very similar to autophagosomes and contain the viral replicase complex [5]. Moreover, NS5A was shown to induce autophagy [27, 28, 30], as was the case for NS4B [26, 29].

**Fig 4:**
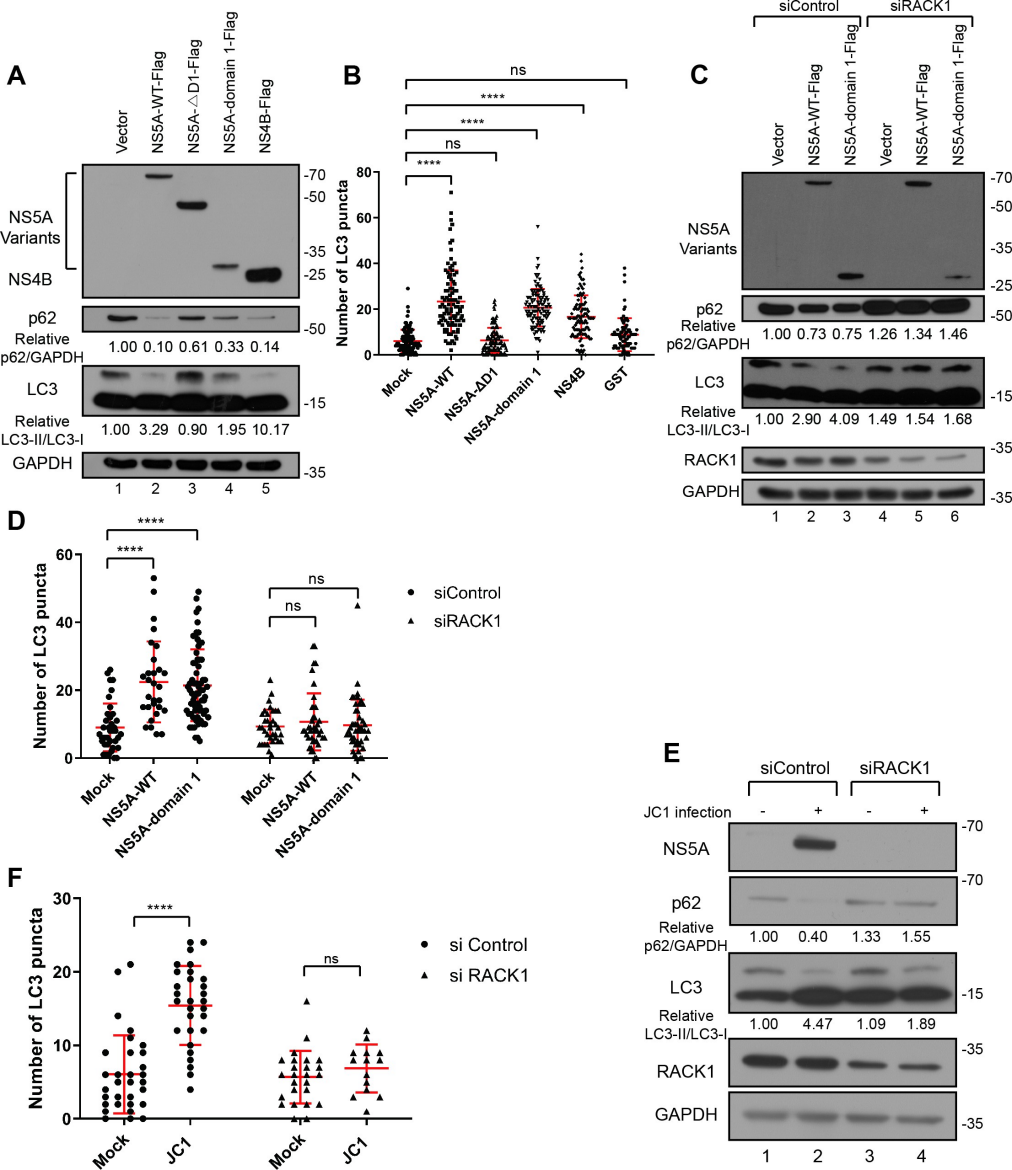
RACK1 is required for the autophagy induction by NS5A. (A) Biochemical analyses of autophagy induction. Huh7.5.1 cells were transfected with plasmids expressing NS4B and NS5A variants. Two days post-transfection, cells were harvested, and the levels of autophagy marker proteins (p62 and LC3) were monitored by Western blotting. The relative levels of p62, which were normalized by GAPDH, are given below each lane. The p62 level in control vector-transfected cells is set to 1. In the case of LC3, the relative ratios of LC3-II to LC3-I are given below each lane. The ratio of LC3-II to LC3-I in control vector-transfected cells is set to 1. At least 3 independent experiments were performed. (B) Number of LC3 puncta per cell. At least 70 cells were analyzed for each set. Huh7 cells expressing GFP-LC3 (GFP-LC3 Huh7 cells) were used in LC3 puncta formation assays. NS4B or NS5A variants were expressed by using a pWPI-based lentivirus system. The lentiviruses were inoculated to GFP-LC3 Huh7 cells and cultivated overnight. The cells were further cultivated for 48 h after changing the media. The cells were fixed and analyzed by an immunocytochemical method. P value was less than 0.0001 by the analysis with ordinary one-way ANOVA multiple comparisons. ns stands for no significance. (C-D) Effects of RACK1 knockdown on the autophagy induction by NS5A. (C) Biochemical analyses of autophagy induction. Huh7.5.1 cells were transfected with a RACK1-specific siRNA. One day later, plasmids encoding NS5A-WT or NS5A-domain 1 were transfected into these cells. Two days later, cells were harvested, and the abundance of autophagy marker proteins (p62 and LC3) was monitored by Western blotting. The relative levels of p62, normalized to GAPDH, are given below each lane. The p62 level in control siRNA- and vector-transfected cells is set to 1. The relative ratios of LC3-II to LC3-I are depicted in numbers. The ratio of LC3-II to LC3-I in control siRNA- and vector-transfected cells is set to 1. 3 independent experiments were performed. (D) Number of LC3 puncta per cell. At least 29 cells were analyzed for each set. GFP-LC3 Huh7 cells were transfected by RACK1 siRNA. One day post-transfection, lentiviruses expressing either NS5A-WT or NS5A-domain 1 were inoculated to the cells. 48 hours after infection, cells were fixed, and samples were analyzed by immunocytochemistry. P value was less than 0.0001 by the analysis with ordinary one-way ANOVA multiple comparisons. ns stands for non-significant difference. (E-F) Effects of RACK1 knockdown on the autophagy induction upon HCV infection. (E) Huh7.5.1 cells were transfected by siRNAs against RACK1. One day post-transfection, the cells were inoculated with JC1 (0.1 MOI). After 4 h of incubation, the media were changed to fresh media. Two days after infection, the levels of autophagy marker proteins (p62 and LC3) were monitored by Western blotting. The relative levels of p62, which were normalized by GAPDH, are depicted in numbers. The p62 level in control siRNA-transfected and mock-infected cells is set to 1. The relative ratios of LC3-II to LC3-I are depicted in numbers. The ratio of LC3-II to LC3-I in control siRNA-transfected and mock-infected cells is set to 1. 3 independent experiments were performed. (F) Number of LC3 puncta per cell. GFP-LC3 Huh7 cells were transfected with RACK1-targeting siRNAs. On the next day, cells were infected with HCV (isolate JC1). 48 hours after HCV infection, cells were fixed and analyzed by immunofluorescence microscopy. At least 14 cells were analyzed for each set. P value was less than 0.0001 by the analysis with ordinary one-way ANOVA multiple comparisons. ns stands for non-significant difference.

In the initial set of experiments, we monitored biochemical changes occurring during autophagy process such as LC3 conversion and degradation of p62 by using Western blotting after ectopic expression of either NS5A or NS4B (Fig 4A). The ratio of LC3-II to LC3-I, which is an indication of autophagy, increased upon ectopic expression of NS5A-WT, NS5A-D1, or NS4B proteins, but with NS5A-ΔD1. Moreover, the level of p62 decreased dramatically when NS5A, NS5A-domain 1, or NS4B protein were ectopically expressed, but only weakly when NS5A-ΔD1 protein was expressed (Fig 4A).

To further confirm the autophagy induction by these proteins, we used GFP-LC3 cells in which LC3 puncta are generated when autophagy is induced [36]. The number of LC3 puncta in the cell was increased when NS5A-WT, NS5A-domain 1, or NS4B protein was expressed ectopically, but with NS5A-ΔD1 protein (Fig 4B and S4 Fig). These results indicate that both NS4B and domain 1 of NS5A induce autophagy.

We investigated the requirement of RACK1 in the NS5A-mediated autophagy induction by a biochemical method (Fig 4C) and a microscopy approach (Fig 4D and S3 Fig). As expected, the level of p62 decreased when NS5A and NS5A-domain 1 proteins were expressed ectopically in control siRNA-treated cells (compare lanes 2 and 3 with 1 in Fig 4C). Similarly, the ratio of LC3-II to LC3-I increased when NS5A-WT and NS5A-domain 1 proteins were expressed ectopically in control siRNA-treated cells (compare lanes 2 and 3 with 1 in Fig 4C). In contrast, the changes in the level of p62 and the ratio of LC3-II to LC3-I mediated by the expressions of NS5A-WT and NS5A-domain 1 proteins were abolished by the RACK1 knockdown (compare lanes 5 and 6 with 4 in Fig 4C). Consistent with these results, the knockdown of RACK1 abolished LC3 puncta formation induced by NS5A-WT and NS5A-domain 1 proteins (Fig 4D and S5 Fig). These results suggest that RACK1 participates in the autophagy induced by the NS5A domain 1.

Furthermore, we investigated whether RACK1 is necessary for the induction of autophagy triggered by HCV infection, using a biochemical method (Fig 4E) and a microscopy approach (Fig 4F and S6 Fig). HCV infection induced autophagy in control siRNA treated cells as shown by the reduction of p62 and the increased ratio of LC3-II to LC3-I (Fig 4E, compare lane 2 with 1). In contrast, RACK1 knockdown abolished both the reduction of p62 and the increase of LC3-II to LC3-I ratio induced by HCVcc (JC1) infection (compare lane 4 with 3 in Fig 4E). Consistently, the number of LC3 puncta significantly increased in the HCV-infected cells when a control siRNA was treated, but that remained the same in the HCV-infected cells when RACK1 was depleted (Fig 4F and S6 Fig). These results indicate that RACK1 is required for HCV-mediated autophagy induction.

### NS5A interacts with ATG14L-Beclin1-Vps34-Vps15 vesicle nucleation complex

**Fig 5:**
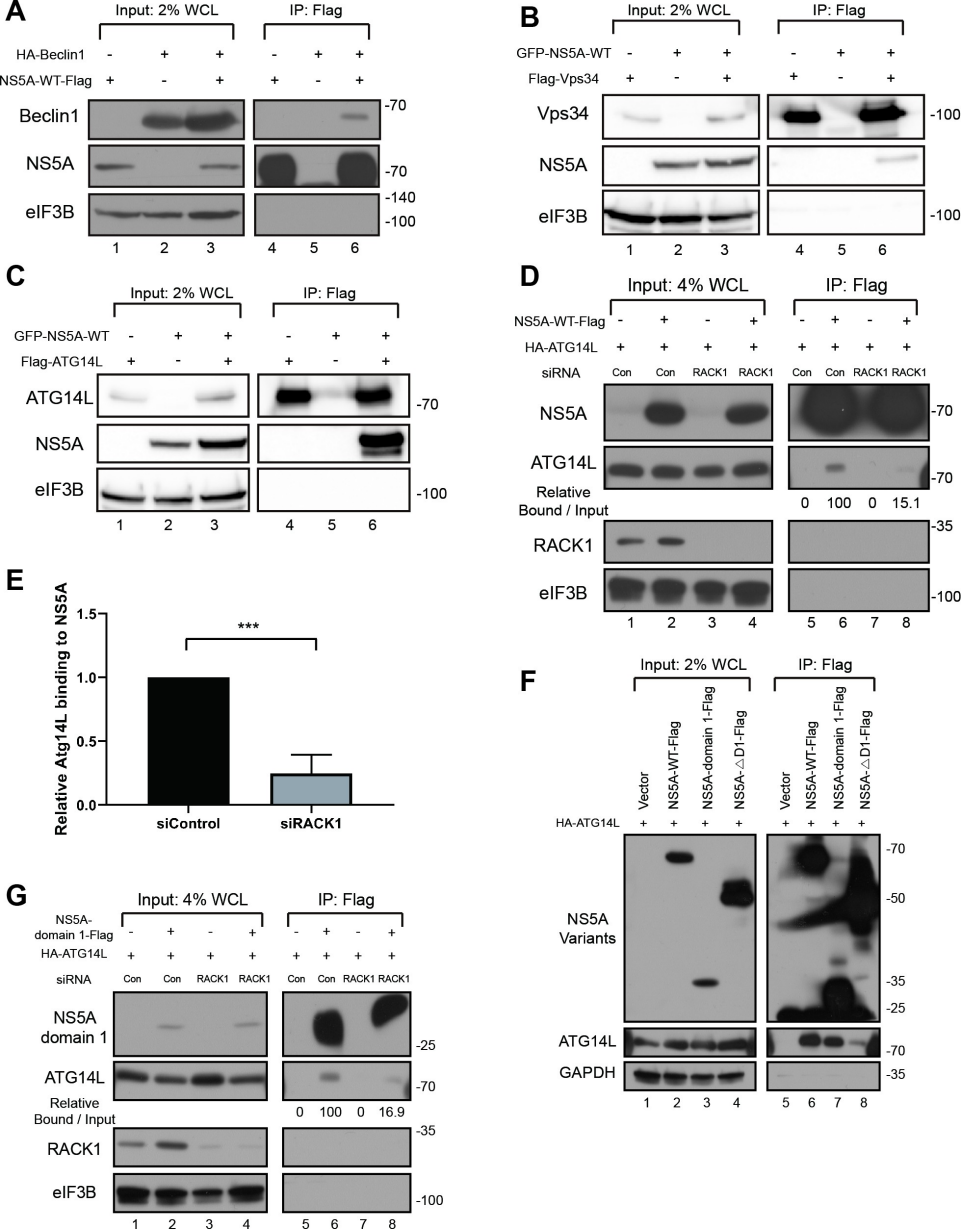
NS5A interacts with ATG14L-Beclin1-Vps34-Vps15 complex. (A) Plasmids encoding Flag-tagged NS5A-WT and HA-tagged Beclin were co-transfected into HEK293FT cells. 48 hours post-transfection, pulldown experiments were performed with a Flag-resin, and the resin-bound proteins were analyzed by Western blotting. 2% of Flag-captured proteins were loaded onto the gel. WCL, whole cell lysate. (B, C) Plasmids encoding Flag-tagged Vps34 or ATG14L was co-transfected with GFP-tagged NS5A-WT into HEK293FT cells. 48 hours post-transfection, pulldown experiments with Flag-resin, and resin-bound proteins were by Western blotting. 2% of Flag-captured proteins were loaded onto the input lanes. WCL, whole cell lysate. (D-E) Effect of RACK1 knockdown on NS5A-ATG14L interaction. RACK1 siRNA was transfected to Huh7 cells. One day after the siRNA transfection, plasmids encoding NS5A-Flag and HA-ATG14L were co-transfected into the cells. Two days after the DNA transfection, the ATG14L-NS5A interaction was analyzed by Flag-resin precipitation and Western blotting. 4% of Flag-captured proteins were loaded onto the input lanes. WCL, whole cell lysate. (E) Band intensities of 3 independent experiments were quantified and depicted by bar graphs. ***, P value < 0.001 by unpaired, two-tailed Student’s t-test. (F) Determination of the ATG14L-binding region in NS5A. Plasmids encoding HA-ATG14L and Flag-tagged NS5A variants were co-transfected into HEK293FT cells. 48 hours post-transfection, pulldown experiments were performed with a Flag-resin, and resin-bound proteins were analyzed by Western blotting. 2% of Flag-captured proteins were loaded onto the input lanes. WCL, whole cell lysate. (G) Effect of RACK1 knockdown on the interaction between ATG14L and NS5A-domain 1. siRNA against RACK1 was transfected into Huh7 cells. One day after the siRNA transfection, plasmids encoding NS5A-domain 1-Flag and HA-ATG14L were co-transfected into the cells. Two days after the DNA transfection, the interaction between ATG14L and NS5A-domain 1 was analyzed by Flag-resin precipitation and Western blotting. 4% of Flag-captured proteins were loaded onto the input lanes. WCL, whole cell lysate.

Since RACK1 was shown to be a key mediator required for the autophagy induction by the vesicle nucleation complex [15, 37], we performed co-immunoprecipitation experiments to decipher the molecular basis of autophagy induction by NS5A and RACK1. HEK293FT cells were transfected with various NS5A constructs and tagged Beclin1 or Vsp34 or ATG14L or Vps15 constructs and captured protein complexes were analyzed by Western blotting. We found that NS5A-WT was co-precipitated with Beclin1, Vps34, and ATG14L (Fig 5A–5C), but not with Vps15 (S7A Fig). This result indicates that distinct components of the vesicle nucleation complex interact with HCV NS5A directly or indirectly.

Next, we investigated the effects of RACK1 knockdown on the interaction between NS5A and proteins of vesicle nucleation complex. The interaction between NS5A-WT and ATG14L was dramatically reduced after partial depletion of RACK1 (Fig 5D and 5E). In contrast, the interactions of NS5A-WT with Beclin1 and with Vps34 were not affected by the RACK1 knockdown (S7B and S7C Fig). By using the same co-immunoprecipitation method, we found that the NS5A domain 1, which binds to RACK1, was required and sufficient for the interaction with ATG14L (Fig 5F, compare lane 7 with 8). Importantly, RACK1 was required for the interaction between domain 1 of NS5A and ATG14L (Fig 5G, compare lane 6 with 8). Note that the residual co-precipitation of ATG14L with NS5A observed in RACK1 knockdown cells (lane 8 in Fig 5D and 5G) is most likely attributable to only the partial knockdown of RACK1. In summary, these results suggest that NS5A interacts with ATG14L in the vesicle nucleation complex in a RACK1 and NS5A-D1 dependent manner.

### ATG14L is required for HCV replication

**Fig 6:**
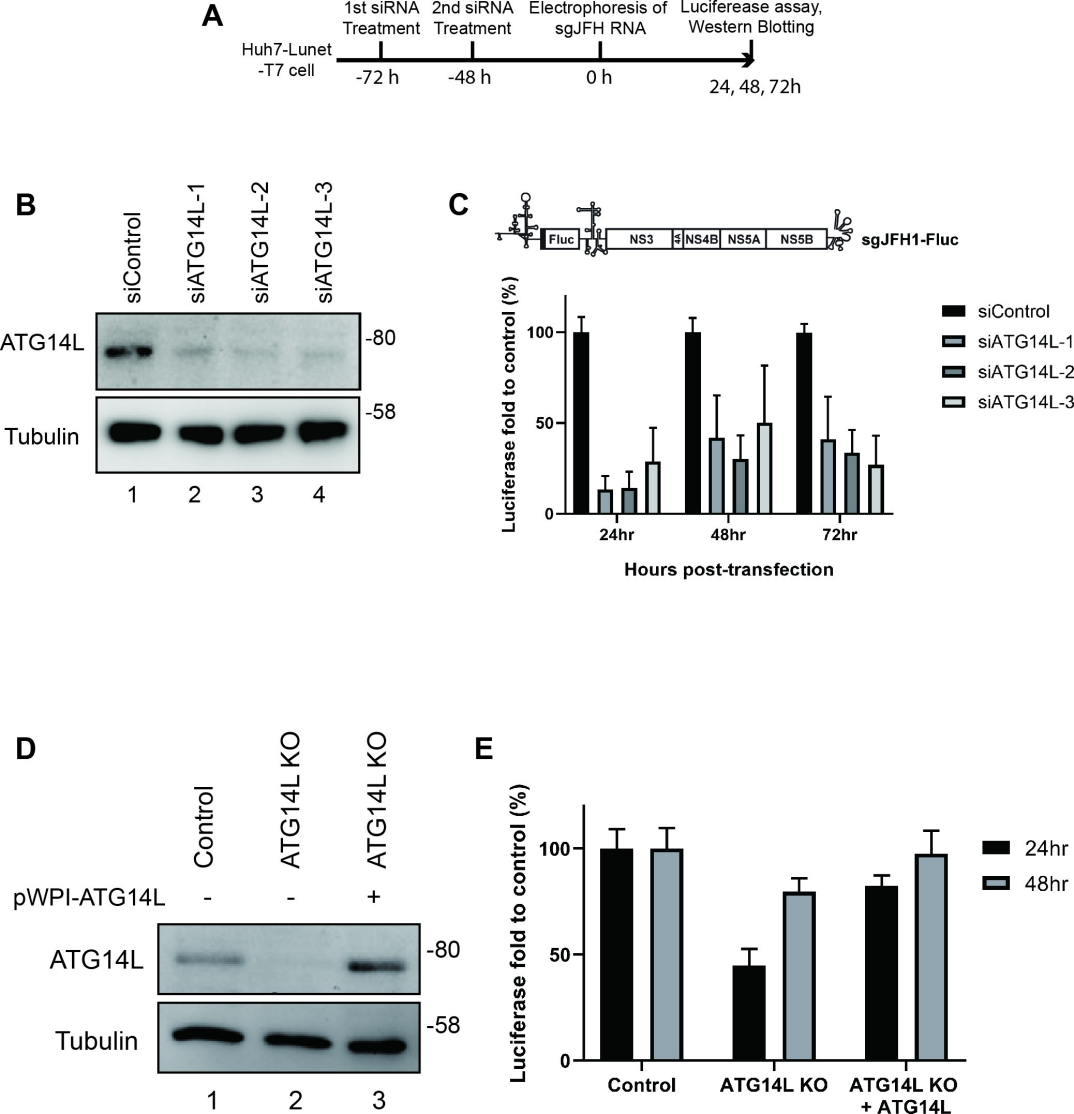
ATG14L are required for HCV replication. (A-C) The effects of ATG14L knockdown on HCV replication. (A) Huh7-Lunet/T7 cells were treated with 3 different siRNAs against ATG14L twice, and a subgenomic replicon RNA containing firefly luciferase gene (sgJFH1-Fluc, [8, 38]) was transfected into the cells 72 h after the first siRNA treatment. (A) Experimental schedule of ATG14L knockdown and RNA replicon transfection. (B) The protein levels of ATG14L and tubulin were monitored by Western blotting 48 h after the replicon RNA transfection. (C) Firefly luciferase activities were measured at 24, 48, and 72 h after the transfections of the replicon RNA. Relative luciferase values were normalized to the values at 4 h after replicon RNA transfection reflecting transfection efficiency. (D-E) The effect of ATG14L knockout on HCV replication. ATG14L-knockout Huh7-Lunet/T7 cells were generated to investigate the role of ATG14L in HCV replication. Control cells (Control), ATG14L-knockout cells (ATG14L KO), and ATG14L-knockout cells infected with lentiviruses expressing ATG14L resistant to sgRNA (ATG14L KO + ATG14L) were transfected with sgJFH1-Fluc and then harvested at 4, 24, and 48 h after replicon RNA transfection. (D) ATG14L levels were monitored by Western blotting using the cell extracts prepared at 48 h after sgJFH1-Fluc RNA transfection. Tubulin served as a loading control. (E) Luciferase activities in the cell extracts were measured, and relative luciferase values were normalized to the values 4 h after replicon RNA transfection reflecting transfection efficiency.

We examined the effects of ATG14L knockdown on HCV RNA replication by using three different siRNAs targeting ATG14L and the subgenomic HCV replicon sgJFH-Fluc [8, 38]. Replication of this RNA was dampened by ATG14L knockdown with each of the siRNAs at all time points after transfection of the replicon RNA (Fig 6A–6C) arguing that ATG14L plays a vital role in HCV RNA replication. We confirmed the role of ATG14L in HCV RNA replication using an ATG14L-knockout Huh7-Lunet/T7 single cell line generated by a CRISPR-Cas9 system (Fig 6D). We also investigated the effect of ATG14L ectopic expression in the ATG14L-knockout cells (rescue experiments) on HCV RNA replication. ATG14L knockout reduced the replication of HCV especially 24 h post-transfection of the replicon RNA (Fig 6E, columns ATG14L KO). In contrast, the ectopic expression of ATG14L nullified the ATG14L-knockout effect on HCV RNA replication (Fig 6E, columns ATG14L KO + ATG14L). Taken together, we concluded that ATG14L is required for efficient HCV RNA replication.

### Both RACK1 and ATG14L are required for NS5A-mediated DMV formation

**Fig 7:**
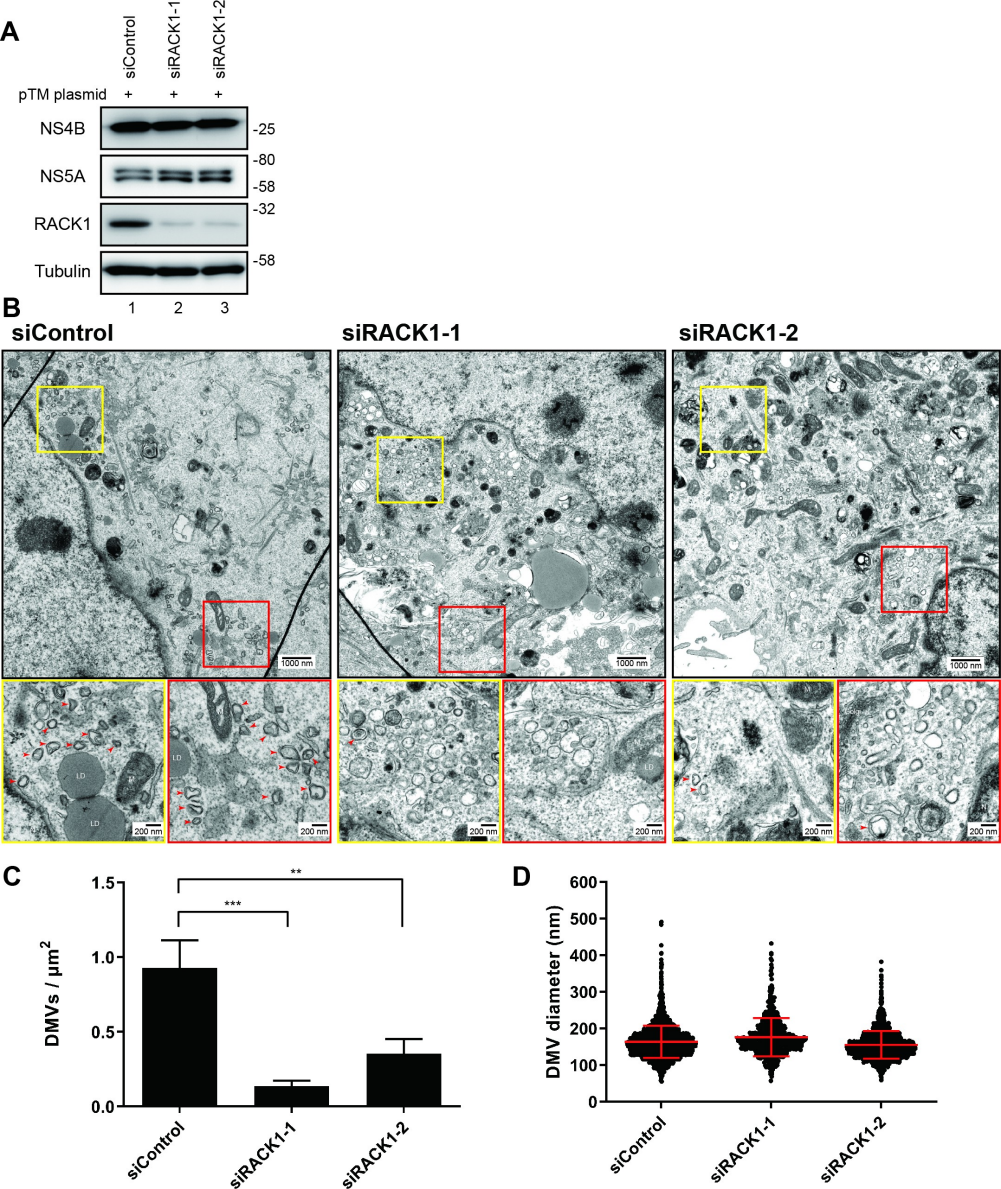
RACK1 is important for DMV formation. (A-D) RACK1 is required for DMV formation. Huh7-Lunet/T7 cells were transfected with two different RACK1 siRNAs twice in 24 h interval. 48 hours after the second siRNA transfection, the cells were transfected with the pTM expression vector encoding the HCV NS3-5B polyprotein, and 24 h later, cells were harvested. (A) Protein expression levels were detected by Western blotting using indicated antibodies. (B-D) RACK1 knockdown cells transfected with pTM plasmid were fixed, processed, and analyzed by TEM. (B) Representative EM images of a total of 772 images. Yellow and red boxes in the overviews indicate regions that are magnified in the lower panels. Upper and lower scale bars indicate 1000 nm and 200 nm, respectively. HCV-induced DMVs are depicted by red arrowheads. LD, lipid droplet; M, mitochondrion; N, nucleus. (C) Quantification of DMV number. DMVs within a whole cell section were counted and divided by cell area (μm^2^). Graph shows average and SD from 10 different DMV-positive cells for each sample. Results were analyzed using the ordinary one-way ANOVA with multiple comparisons. p < 0.01 (**) and p < 0.001 (***) values are considered statistically significant. (D) Quantification of DMV diameter. 2637, 601, or 1475 DMVs from 10 cells in each sample were analyzed for control, siRACK1-1, or siRACK1-2, respectively.

As described above, autophagy induction and DMV formation triggered by NS5A are required for HCV RNA replication. Having found that RACK1 is required for autophagy induction (Fig 4), NS5A binding to the vesicle nucleation complex (Fig 5) and HCV RNA replication (Fig 2) we investigated whether RACK1 is required for HCV-mediated DMV formation. For this purpose, we used a cytoplasmic expression system in which the expression of the HCV NS3-5B polyprotein induces the DMV formation [9]. To this end, Huh7-Lunet/T7 cells were transfected with control or RACK1 siRNAs and transfected with the pTM NS3-3’UTR expression plasmid (Fig 7A). DMV formation in the cells was monitored by transmission electron microscope (TEM) (Fig 7B). In RACK1 knockdown cells, the number of DMVs was profoundly reduced as compared with control cells (Fig 7C), whereas DMV size was not affected (Fig 7D).

**Fig 8:**
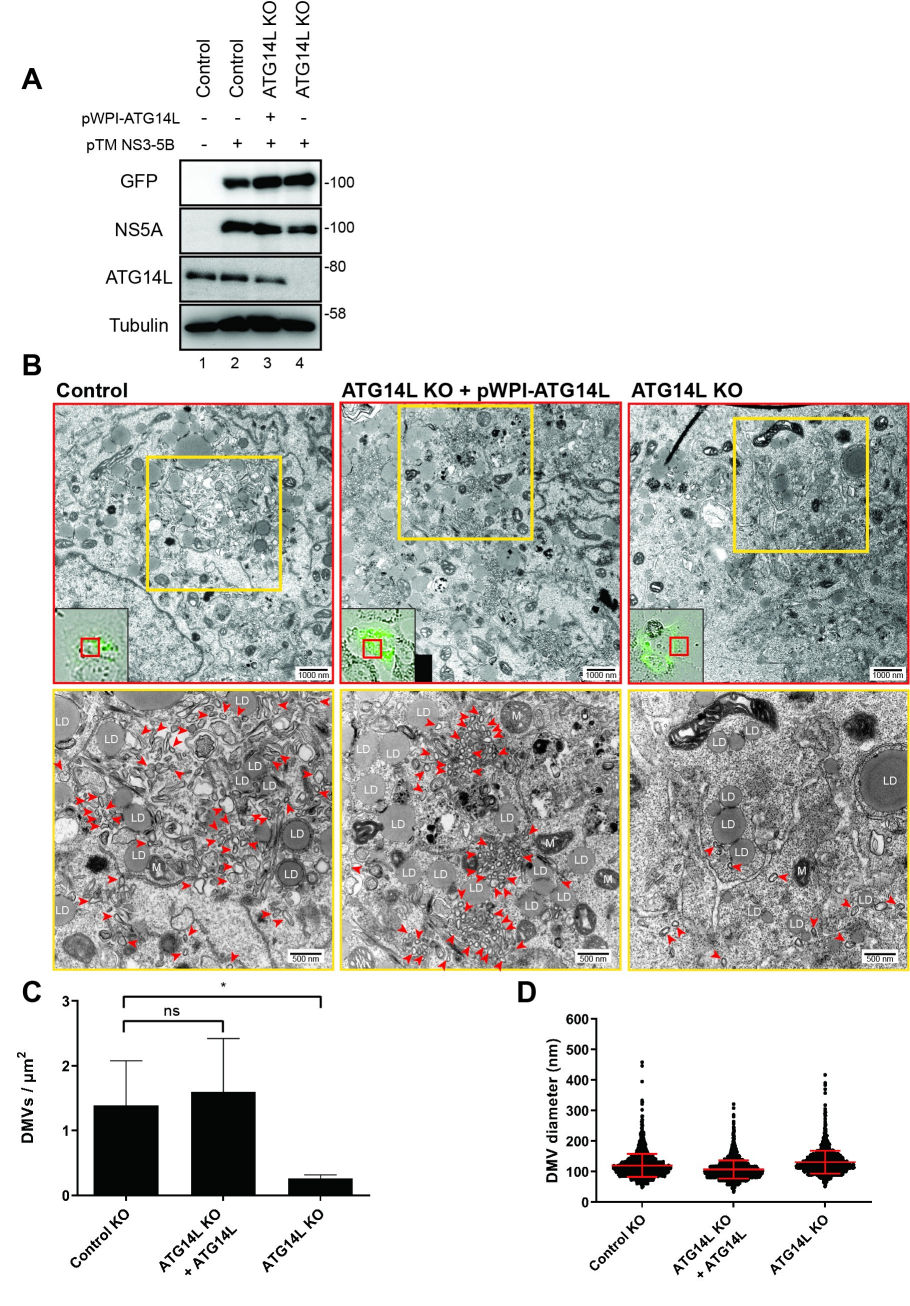
ATG14L is required for DMV formation. (A-D) Effects of ATG14L knockout on DMV formation. Control, ATG14L KO + ATG14L, and ATG14L KO cells (refer to Fig 6D and 6E) were transfected with pTM plasmid encoding the NS3-5B polyprotein with NS5A containing an in-frame GFP insertion. Cells were analyzed 24 h post-transfection. (A) Protein levels were detected by Western blotting using corresponding antibodies. (B-D) Cells transfected with the NS3-5B expression plasmids were fixed, processed, and analyzed by CLEM. (B) Representative images highlighting HCV-induced DMVs (red arrowheads). Low resolution confocal microscopy images identifying transfected cells via gfp fluorescence are depicted in the bottom left boxes in the top panels. Yellow boxes in the top panels are magnified in the corresponding bottom panels. DMVs within a whole cell section were counted and divided by cell area (μm^2^). LD, lipid droplet; M, mitochondrion. Upper and lower scale bars indicate 1000 nm and 500 nm, respectively. (C) Quantification of DMV number. Total 4, 6, or 7 GFP-positive cells were analyzed for control, ATG14L KO + ATG14L, or ATG14L KO, respectively. Graphs show average and SD. Each data set was compared with control cells. Results were analyzed using the ordinary one-way ANOVA with multiple comparisons. p < 0.05 (*) value is considered statistically significant. ns stands for non-significant difference. (D) Quantification of DMV diameter. 2005, 3517, or 1373 DMVs in each sample were analyzed for control, ATG14L KO + ATG14L, or ATG14L KO, respectively.

We also investigated the role of ATG14L in DMV formation by using ATG14L KO cell and Huh7-Lunet/T7 as parental cell line (Fig 8A). The plasmid pTM NS3-3’UTR containing GFP in the NS5A region was transfected into ATG14L KO cells or ATG14L KO cells transfected with pWPI-ATG14L (ATG14L KO + pWPI-ATG14L). DMV formation was monitored by correlative light and electron microscopy (CLEM) 24 h after DNA transfection (Fig 8A and 8B). In the ATG14L KO cells, the number of DMVs was profoundly reduced as compared with control cells (Fig 8B and 8C) whereas DMV size was not affected (Fig 8D). The reduction of DMV number in ATG14L KO cells were completely abolished by ectopic expression of ATG14L in ATG14L KO cells (Fig 8B and 8C, ATG14L KO + pWPI-ATG14L). These results indicate that both RACK1 and ATG14L play critical roles in the robust HCV-mediated establishment of the HCV replication organelle.

## Discussion

Although previous studies have reported that the autophagy process and DMV formation are required for HCV RNA replication [39], the molecular mechanisms underlying membrane rearrangement induced by HCV infection remained mostly unknown. To gain insights into the molecular mechanism of HCV replication, we sought to identify cellular proteins facilitating the HCV proliferation by the yeast two-hybrid system using the core, NS3 and NS5A genes of HCV as baits. Through this screening, 32 cellular proteins were revealed as potential candidates interacting with HCV proteins. The demand for RACK1 in HCV replication was the highest one among cellular proteins tested by knockdown experiments (Fig 1).

RACK1 was reported to interact with the components of vesicle nucleation complex composed of Beclin1, ATG14L, Vps34, and Vps15 during autophagy process [15]. RACK1 directly interacts with Beclin1, ATG14L and Vps15 and indirectly with Vps34 when AMP-activated protein kinase (AMPK) phosphorylates the Thr50 of RACK1 under starvation conditions [15]. RACK1 supports the vesicle nucleation complex assembly [i.e., the linkage between the ATG14L-Beclin complex with Vps15-Vps34 complex [16, 37]] that activates phosphoinositide 3-kinase (PI3K) activity of Vps34 resulting in autophagy induction [15].

**Fig 9:**
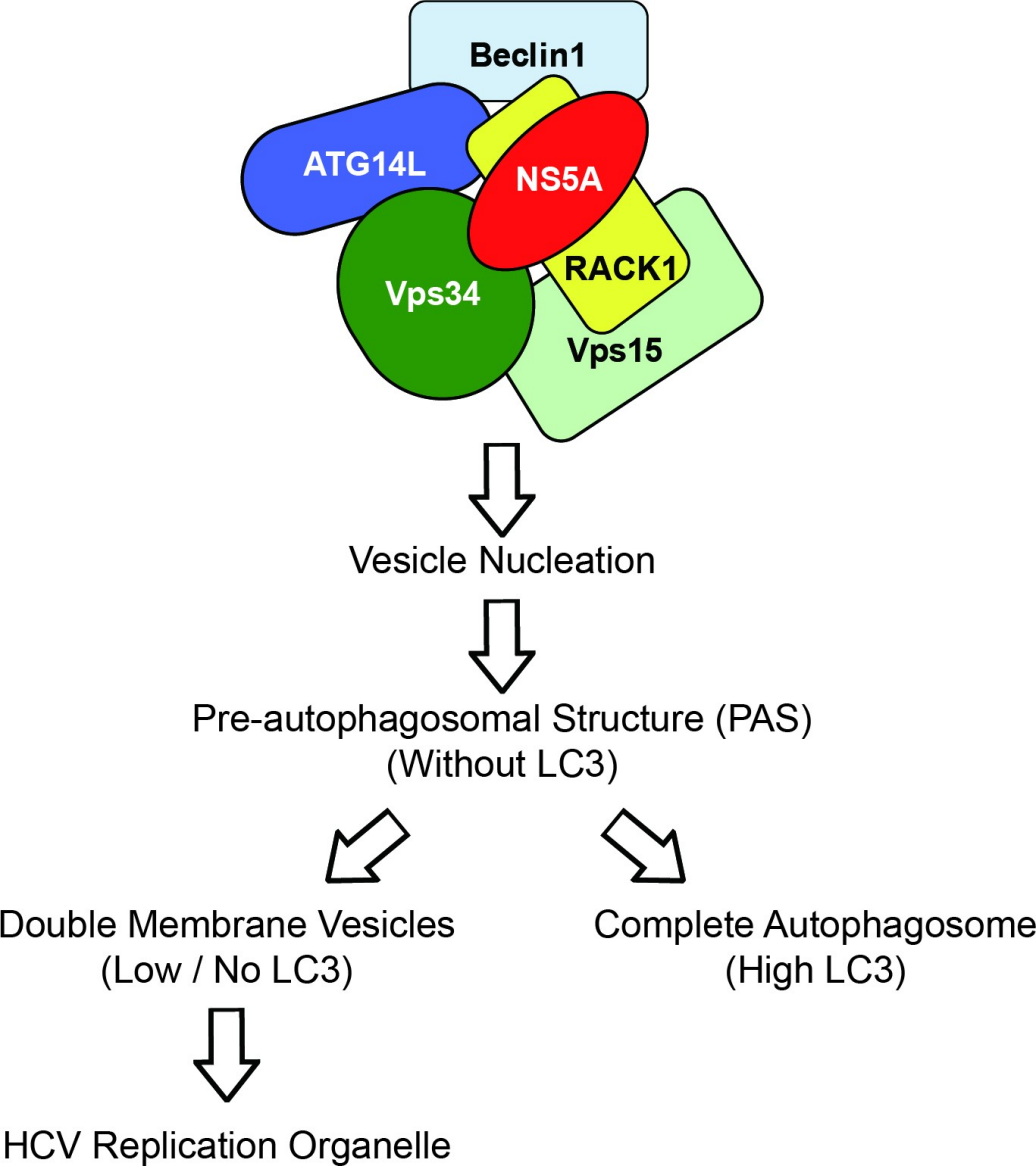
Schematic diagram of the vesicle nucleation complex induced by HCV NS5A. A schematic protein-protein interaction model of vesicle nucleation complex induced by NS5A. According to our data, NS5A interacts with Vps34 and Beclin1 directly or indirectly. Moreover, NS5A interacts with ATG14L with the help of RACK1. These interactions seem to facilitate the formation of vesicle nucleation complex composed of ATG14L-Beclin1-Vps34-Vps15 by bridging Beclin1-ATG14L complex with Vps34-Vps15 complex. The vesicle nucleation complex, in turn, triggers the formation of pre-autophagosomal structure (PAS) composed of ATG5, ATG12, and ATG16L1, which induces vesicle expansion. We speculate that the PAS has two alternative fates even though the underlying molecular mechanisms remain unknown (see Discussion): 1) The PAS is used in the construction of replication organelles via DMV formation. This process may not require LC3. 2) The PAS is used in the construction of complete autophagosomes. This process requires the conversion of LC3-I to LC3-II.

Here we report that HCV NS5A induces autophagy and DMV formation with the help of RACK1 (Figs 4 and 7). We also show that NS5A directly or indirectly interacts with Vps34 and Beclin1 and associates with ATG14L in the presence of RACK1 (Fig 5). Considering that RACK1 facilitates the formation of vesicle nucleation complex under certain physiological conditions such as starvation, we propose that an active vesicle nucleation complex is formed by NS5A with the help of RACK1. The interaction of Beclin1 and Vps34 with NS5A may play a key role in linking the Beclin1-ATG14L complex with the Vps34-Vps15 complex for induction of vesicle nucleation. The NS5A-RACK1 interaction and the interactions of RACK1 with Beclin1, ATG14L and Vps15 seem to augment the formation of the vesicle nucleation complex and/or to facilitate proper positioning of the components of the complex (Fig 9). The knockdown experiments with three different siRNAs targeting ATG14L mRNAs (Fig 6C) as well as the knockout and rescue experiments of ATG14L gene (Figs 6E and 8) demonstrated that ATG14L plays a vital role in the construction of HCV replication organelles. Nevertheless, we observed that HCV RNA replication occurs in the absence of ATG14L, despite an absolute delay in the replication kinetics under this condition (Fig 6E). The result suggests that ATG14L is required for efficient HCV replication, but an alternative pathway of autophagy induction, bypassing the requirement of ATG14L, might have been activated during the establishment of ATG14L-knockout cells, which results in the construction of HCV replication organelles albeit inefficiently in the ATG14L-knockout cells. In this respect, it is worth to note that HCV RNA replication was not recovered even at 72 h after transfection of replicon RNA in ATG14L-knockdown cells (Fig 6C).

Recently, it was reported that HCV infection induces ATG5- and ATG14-dependent selective autophagy. Interestingly, the HCV-mediated autophagy is independent of ATG13 [40], arguing that HCV-induced DMV formation bypasses the autophagy induction complex composed of ATG13, ATG101, ULK1 and FIP200 [16]. Considering our study and previous reports, we propose that HCV NS5A induces DMV formation by hijacking cellular proteins participating in the early (vesicle nucleation) stage of autophagy.

A recent study reported that components of the pre-autophagosomal structure (PAS; ATG5, ATG12, and ATG16L1), which induces vesicle expansion [16, 17, 41], co-precipitated with HCV NS4B that was associated with DMVs isolated from HCV replicon-containing cells [39]. In contrast, LC3-I or LC3-II was not associated with NS4B-containing DMVs. Moreover, the knockdown of either ATG7 or ATG12 inhibited DMV formation in cells continuously expressing HCV nonstructural proteins, whereas LC3 knockdown did not affect DMV formation [39]. The results suggest that the vesicle expansion step of autophagy, but not the vesicle completion step demanding LC3-II, is required for the DMV formation. Interestingly, NS4B, which also induces autophagy in Huh7 cells (Fig 4; also see [29]), seems to participate in DMV formation at the vesicle expansion step plausibly by interacting with PAS proteins. However, it is not clear whether other HCV proteins such as NS5A and NS5B also participate in the vesicle expansion step since these viral proteins are also present in the NS4B-containing DMVs [39]. It is noteworthy that other HCV proteins do not induce DMV formation in the absence of the NS5A domain 1 [8] and that NS5A protein alone, albeit weaker, can induce DMV formation [9]. It remains elusive how the autophagy components for vesicle expansion communicate with HCV proteins for DMV formation.

It was shown that autophagosome formation occurs during HCV infection and in NS4B and NS5A expressing cells [26–29]. A resent study showed that NS5A expression leads to an increase in autophagy flux [30]. Autophagosome formation is thought to reduce innate immune responses by degrading innate immune signaling proteins, such as MAVS and ISG56 [22, 24, 25]. It remains elusive how the DMV and autophagosome formations are controlled.

Although RACK1 is a constituent of the eukaryotic ribosome, at least under our experimental conditions, it did not affect HCV IRES-dependent translation (Fig 2D–2F, S2 Fig). This result is consistent with the ones obtained by genome-wide screening indicating that RACK1 is required for HCV replication but not for HCV IRES-dependent RNA translation [19]. However, these results are inconsistent with a report by Majzoub and colleagues suggesting that RACK1 is required for the activity of the HCV IRES [42]. This variance might be due to the use of different experimental conditions, but further analyses are required to clarify this discrepancy.

It is noteworthy that the endogenous RACK1 was not detected in the co-immunoprecipitation experiments using NS5A variants shown in Fig 5D and 5G. The result is likely due to the multifunctional activities of RACK1. In fact, RACK1 has more than 100 known interaction partners with the majority of RACK1 proteins residing in the cytosol as a component of the 40S ribosome [13, 14]. Therefore, only a minor portion of RACK1 proteins are likely to participate in autophagy induction. We confirmed the interaction between NS5A and endogenous RACK1 in NS5A immune-complexes isolated from NS5A-expressing cells (S8A Fig) or RACK1 complexes isolated from Huh7 cells containing a HCV replicon (S8B Fig).

Consistent with a previous report [43], YWHAE (also called 14-3-3) was identified as a Core-binding protein by using the two-hybrid screening (S1 Table). This indicates that our screening is valid. Another potential partner of interest is NSUN5 that is likely to interact with Core (S1 Table). NSUN5 is an m^5^C-RNA methyltransferase. According to a recent article, post-transcriptional modification (PTM) of cytosine(s) in HCV RNA to m^5^Cm is required for proliferation of HCV even though the enzyme responsible for this modification is not known [44]. Considering the function of NSUN5 and its potential partner Core, investigations into the role of NSUN5 in HCV proliferation in conjunction with Core protein may help to reveal the molecular basis of PTM functions in HCV proliferation.

In conclusion, we report that the components of vesicle nucleation complex, acting at the early stage of autophagy, participate in DMV formation through interactions with NS5A and/or RACK1. The interaction between RACK1 and NS5A domain 1 augments the DMV formation, which is the main component of the HCV replication organelle. These results contribute to our understanding of the molecular mechanisms underlying the formation of replication organelles of HCV and other positive-strand RNA viruses.

## Materials and methods

### Antibodies and reagents

NS5A antibodies were kindly provided by Dr. Charles Rice (Clone 9E10, mouse monoclonal antibody), and Dr. Byung Yoon Ahn (rabbit polyclonal antibody). NS4B antibody was generated from rabbit [12]. RACK1 antibodies were purchased from Abcam (ab62735), BD sciences (610177), and Santa Cruz (sc-17754). The antibodies against NS5B (sc-17532), HA (sc-7392, sc-805), GFP (sc-9996), p62 (sc-28359), and eIF3B (sc-16377) were purchased from Santa Cruz. Flag antibodies (F3165, F7425), and Tubulin antibody (T6074) were purchased from Sigma Aldrich. The antibodies against LC3 (#2775), and PI4KA (#4902) were bought from Cell Signaling. ATG14L antibody (PD026), Actin (0869100), GAPDH antibody (4G5) were purchased from MBL, MP Biomedicals, and AbD Serotec. For Western blotting, sheep HRP-anti-mouse IgG (NA931V; GE Healthcare), donkey HRP-anti-rabbit IgG (NA934; GE Healthcare), and rabbit HRP-anti-goat IgG (81–1620; Invitrogen) antibodies were used as secondary antibodies. For immunocytochemical experiments, goat cyanine 5 anti-mouse IgG (A10524; Invitrogen), and goat Alexa Fluor 555 anti-rabbit IgG (Z25305; Invitrogen) were used as secondary antibodies. Hoechst33258 (H3569; Invitrogen) was used for nucleus staining.

FugeneHD (Promega), Lipofectamin3000 (Invitrogen), Oligofectamin (Invitrogen), Lipofectamine RNAiMAX (Thermo Fisher) and TransIT-LT1 (Mirus Bio) reagents were used for DNA transfection. Anti-Flag M2 Magnetic beads (M8823; Sigma Aldrich) were used for immunoprecipitation. Luciferase assay system (E1960; Promega) was used for luciferase assay experiments. ToxiLight^TM^ bioassay kit (Lonza), CellTiter-Glo Luminescent Assay kit (Promega) and MTT (Methylthiazolyldiphenyl-tetrazolium bromide; Sigma Aldrich) were used for measuring cell cytotoxicity and viability according to the manufacturer’s instructions.

### Cell culture

Huh7, Huh7.5.1, and Huh7-Lunet/T7 cells are human hepatocyte-derived carcinoma cell lines [8, 45]. HEK293FT cell is originated from human embryonic kidney cells [46]. Huh7, Huh7.5.1, Huh7-Lunet/T7, and HEK293FT cells were grown in Dulbecco’s modified Eagle’s medium (DMEM; Gibco) supplemented with 10% fetal bovine serum (FBS; Hyclone), penicillin (100 U/ml; Sigma Aldrich), and streptomycin (100 μg /ml; Sigma Aldrich). Cells were maintained at 37°C with 5% CO_2_ environment. Huh7 cells containing HCV replicons were grown under the same conditions, with the additional inclusion of the antibiotic G418 (500 μg/ml; AG Scientific) [47]. Huh7 cells expressing GFP-LC3 were cultured under the same conditions, with the additional antibiotic Blasticidin S (3 μg/ml; Sigma Aldrich). GFP-control and GFP-RACK1 cell lines were originated from Huh7 cells. These cells were selected by the antibiotic G418 (500 μg/ml) after transfection of cells with DNAs containing the corresponding genes.

### Virus production and concentration

*In vitro* transcription and RNA electroporation were performed as previously described [48]. Approximately 3 × 10^6^ Huh7.5.1 cells were electroporated with *in vitro*-transcribed RNAs derived from JC1 or JFH1-ad34-5A-Rluc containing the cell culture adapted mutations in the E2 and p7 proteins [45]. The culture supernatants were collected at 3–5 days after electroporation, and naïve Huh7.5.1 cells were infected with the generated viruses. The cell culture media of infected cells were collected at 3–5 days after infection. The HCV-containing supernatants were then filtered through with a 0.45 μm filter, and the filtrate was concentrated with a Vivaspin (100-kD cut-off; Millipore). The concentrated HCV culture medium was loaded onto a 20% sucrose cushion, and HCV particles were collected by ultracentrifugation at 4°C for 4 h at 36,000 rpm (SW41 rotor; Beckman). The viral pellets were resuspended with complete medium. Regarding the transient replication assay, *in vitro* transcripts of HCV subgenomic replicons were generated, purified, and electroporated into Huh7-Lunet/T7 cells. Firefly luciferase activities in cell lysates were measured as previously described [49].

### Lentivirus based protein expression and cell line establishment

Lentivirus system was used for ectopic expression of proteins in GFP-LC3 puncta assays. GFP-LC3 lentivirus was produced by co-transfection of pWPI-GFP-LC3, Gag-pol, and pMD.G into HEK293FT cells [36]. Two days post-transfection, culture supernatants were collected and centrifuged at 2000 rpm for 5 min along with 0.45 μm filtration to remove cell debris. Virus stocks were kept at -70°C with Opti-prep (Sigma Aldrich) solutions. Huh7 and Huh7.5.1 cells were used to make GFP-LC3 cell lines. GFP-LC3 cells inoculated with lentivirus were treated with 4 μg/ml of polybrene overnight (Sigma Aldrich). The cell culture media was changed with a selection media containing 3 μg /ml of Blasticidin S, and then cells were cultivated for 5 days. The GFP-LC3 expression was monitored by Western blotting and immunocytochemistry. GFP-LC3 expressing cells were sorted by FACS in order to enrich GFP-LC3 expressing cells. The top 5–10% of the cells were collected. DNA fragments corresponding to NS5A-WT-Flag, NS5A-ΔD1-Flag, NS5A-domain 1-Flag, NS4B-Flag, and GST-Flag were amplified by PCR using pcDNA-based constructs as templates. The DNA fragments were inserted into a pWPI-puro vector and used for lentivirus production. To construct the ATG14L sgRNA-resistant mutant, four mutations, which do not change amino acids, were introduced to the target sequence of ATG14L sgRNA.

### Generation of KO cell lines by using CRISPR-Cas9 system

Annealed oligonucleotides were inserted into a lentiCRIPSRv2 plasmid (Addgene). Single guide RNAs are transcribed from this vector through an U6 promoter. Lentiviruses were prepared as described previously [49]. In order to generate knockout (KO) cell lines, Huh7-Lunet/T7 cells were infected with respective lentiviruses. The cells were selected in medium containing 3 μg/ml puromycin (Sigma Aldrich) for 1 week. To isolate single KO clones, cell were seeded onto 96 well plates at 0.5 cells per well without antibiotics. After 4–6 weeks, the ATG14L protein expression was analyzed by Western blotting. Single KO clone cells were infected with lentivirus to express non-tagged ATG14L and selected in medium containing 3 μg/ml blasticidin (Sigma Aldrich) for one week. The sgRNA sequence against ATG14L is ‘5´-TCTACTTCGACGGCCGCGAC-3´’.

### Plasmid constructs

The DNA sequences encoding HCV genes (Core, NS3, and NS5A) were amplified from JFH1 HCV genotype 2a cDNA clone [48]. RACK1, Beclin1, ATG14L, Vps34, and Vps15 genes were obtained by PCR using cDNA libraries from human liver tissue or HeLa cell. The amplified genes were cloned into various plasmids (pcDNA3.1, pEGFP-C1, pGBKT7, and pGADT7) depending on the experimental purposes. Plasmids pcDNA3.1-Flag and pcDNA3.1-HA were constructed as previously described [46]. For the construction of NS5A-expressing plasmid, the DNA segment encoding 1–213 aa of HCV genotype 2a was amplified by PCR and inserted into the pcDNA3.1 vector. For the construction of NS5A-°CD1-expressing plasmid, the DNA segments NS5A 1–30 aa and 214–466 aa were amplified separately by PCR, and then the amplified DNAs were ligated together. The ligated DNA was re-amplified by PCR and inserted into the pcDNA3.1 vector. The sequences of all DNAs amplified by PCR were confirmed by sequencing. All NS5A constructs contain a Flag-tag at the C-terminal ends. pTM NS3-5B (NS5A-GFP) was used for CLEM [9]. pFK_i389LucNS3-3′JFH1_δg (genotype 2a) (°CGDD) and pRL-CMV (promega) were used for short-term translation assay [50].

### Western blotting

Cells were lysed in IP buffer (50 mM Tris HCl (pH 7.5), 150 mM NaCl, 1% NP40, 2 mM Sodium orthovanadate, 10 mM NaF, 10 mM β-glycerophosphate, 1 mM PMSF) [15]. Proteins in lysates were resolved by sodium dodecyl sulfate-polyacrylamide gel electrophoresis (SDS-PAGE) followed by transfer to PVDF membrane (Millipore). Horseradish peroxidase (HRP)-conjugated donkey anti-rabbit IgG, sheep anti-mouse IgG, and donkey anti-goat IgG were used as secondary antibodies. Chemiluminescence detection was performed using WEST-ZOL plus (iNtRON) and West Femto Substrate (Thermofisher). X-ray films (Agfa) and LAS (Image Quant; LAS4000) were used to visualize the chemiluminescence signal. 0.1% TBS-T was used for washing and blocking solution preparation, and 0.1% PBS-T was used for LC3B blotting [51, 52].

### siRNA and DNA transfection

Huh7 or Huh7.5.1 cells seeded on 12 well plates (5 × 10^4^ cells/well). Cells were transfected with siRNAs (200 nM final) or DNAs by using oligofectamine reagent or FugeneHD, respectively, according to the manufacturer’s instructions. Cell culture media were changed with fresh media containing FBS 4 h after the treatments. The levels of proteins were monitored by Western blotting 48 or 72 h post-transfection. For ATG14L knockdown, Huh7-Lunet/T7 cells seeded on 6 cm dish were transfected twice with 50 pmole siRNAs using Lipofectamine RNAiMAX Reagent in 24 h intervals according to the manufacturer’s instructions.

Control siRNA (180831) was purchased from Bioneer. Two siRNAs targeting RACK1 are ‘5`-GCCUCUCGAGAUAAGACCAUCAUCAdTdT-3`’, ‘5`-GGAACCUGGCUAACUGCAAGCUGAAdTdT-3`’ [53], ‘5`- UGGCAGAGCUUU ACAAAUAdTdT-3`’, and ‘5`-GGCAGAGCUUUACAAAUAAdTdT-3`’. PI4KA siRNA sequence is ‘5`-GAGCAUCUCUCCCUACCUAUUdTdT-3`’ [54]. ANPEP siRNA sequence is ‘5`-AACGAUCUCUUCAGCACAUCAdTdT-3`’ [55]. AOX1 siRNA sequence is ‘5`-GCCAAUGCCUGUCUGAUUCdTdT-3`’ [56]. BAAT siRNA sequence is ‘5`-CUAUAAGGACAGGUACUAUdTdT-3`’ which was purchased from Bioneer (1011124). CTSB siRNA sequence is ‘5`-GGAUCACUGCGGAAUCGAAdTdT-3`’ [57]. CYP2C8 siRNA sequence is ‘5`-UAAAGAACCUCAAUACUACdTdT-3`’ [58]. ISOC2 siRNA sequence is ‘5`-CUCCCGGAAAUGCAAAUGAdTdT-3`’ which is purchased from Bionner (1075591). ATG14L siRNAs were ordered from Thermo Fisher Scientific: silencer select negative control no. 2 siRNA (4390846), ATG14L-1 (s22527; 5`- GGGAGAGGUUUAUCGACAAdTdT-3`), ATG14L-2 (s22527; 5`- GGGAGAGGUUUAUCGACAAdTdT-3`), ATG14L-3 (s22526, 5`-GCUUUACAGUCGAGCACAAdTdT-3`).

### RACK1 rescue experiment

GFP-control and GFP-RACK1 cell lines were seeded on 12 well plates (5 × 10^4^ cells/well). 1 day after cultivation of cells, siControl, siRACK1-3, or siRACK1-4 was transfected into cells with oligofectamine, and the media were changed 4 h after transfection. The cells were further cultivated for 1 day and then inoculated with JFH1-ad34-5A-Rluc virus. Luciferase assays and Western blotting were performed 48 h after virus infection.

### Immunoprecipitation

HEK293FT cells, cultivated on 100 mm plates coated by poly-L-Lysine (Sigma Aldrich) for 24 h, were transfected with DNAs by Lipofectamine 3000 according to manufacturer’s protocol. Cells were harvested 48 h post-transfection, lysed in IP buffer, and then sonicated. Cell lysates were centrifuged at 12000 rpm for 30 min, and the supernatants were collected. Flag-resin was used to pull-down Flag-tagged proteins. Beads were washed 4 times by IP buffer and boiled with protein sample buffer to elute proteins. In testing the effects of siRNAs against RACK1 on protein-protein interactions, Huh7 cells were treated with siRNAs for 24 h and then transfected with DNAs. The cells were further cultivated for 48 h and then analyzed by Western blotting after immunoprecipitation.

In immunoprecipitation experiments using a RACK1 antibody, Huh7 cells with or without replicon RNAs were lysed by IP buffer and then sonicated. Cell lysates were mixed with protein G agarose beads (Roche) for 1 h. Non-specific beads-binding proteins were removed by centrifugation at 12000 rpm for 15 min, and the supernatants were collected. 1 μg of RACK1 antibody (sc-17754) and control mouse IgG antibody (sc-2025) were used for immunoprecipitation. Protein G agarose beads were washed 3 times with IP buffer and incubated with antibodies for 4 h. The antibody conjugated beads were washed 3 times by IP buffer, and mixed with cell lysates for 2 h. Beads were washed 4 times with IP buffer and boiled with protein sample buffer to elute proteins.

### Testing effects of siRNAs on HCV RNA replication and HCV IRES activity

An HCVcc (JFH1-ad34-5A-Rluc), genotype 1b replicon (NK/R2AN), and genotype 2a replicon (JFH1-Gluc) containing cells were used to investigate the effects of host factors on HCV replication [48, 51]. For the HCVcc infection, Huh7.5.1 cells were treated with different siRNAs for 24 h and then infected with JFH1-ad34-5A-Rluc virus. Cell lysates were obtained 48 h after infection, and *Renilla* luciferase (Promega) activities in the lysates were measured. In the case of replicon, NK/R2AN and JFH1-Gluc cells were transfected with different siRNAs for 72 h, and then luciferase assays and Western blotting were performed. HCV IRES activities were measured by RNA reporter to avoid cryptic promoter issue. Either co-transfection of 2 mono-cistronic mRNAs or transfection of 1 di-cistronic mRNA (Fig 2C) was performed. RACK1 was depleted by treating a siRNA against RACK1 to Huh7 cells for 72 h, and then 500 ng of reporter RNAs were transfected. Cell lysates were collected 4 h after transfection, and dual luciferase assay was performed. Data represent relative ratios of firefly luciferase activities directed by HCV IRES-dependent translation to *Renilla* luciferase activities directed by cap-dependent translation.

### Short-term translation assay

Short-term RNA translation assay was performed as described previously with minor modifications [50]. To prepare RNA reporters, 5 μg of pRL-CMV plasmid (linearized with BamHI (NEB)) in 100 μl reaction mixture containing 20 μl of 5 × rabbit reticulocyte lysate buffer [400 mM Hepes (pH 7.5), 60 mM MgCl_2_, 10 mM spermidin, 200 mM DTT], 12.5 μl of 25 mM NTP solution containing 12.5 mM GTP, 2.5 μl of RNasin (40 U/μl), 0.1 U pyrophosphatase (Sigma Aldrich), 12.5 mM m^7^G cap analog (NEB), and 6 μl of T7 polymerase. The reactions were performed over night at 37°C. Subsequently, the input DNA was removed using RQ1 RNase-free DNase (Promega). The RNA was purified by acid phenol chloroform extraction and isopropanol precipitation. The pellet was washed with 70% ethanol and resuspended in RNase free water.

Huh7.5 cells were transfected with siRNA twice in 24 h interval. 3 days post-transfection, 3 x 10^6^ cells were suspended in 200 μl (total volume) of Cytomix and electroporated (0.166 kV, 950 μF, 0.2 cm cuvettes) with 5 μg of reporter replicon RNA (ΔGDD) and 5 μg of a capped *Renilla* transcript. Replication deficient ΔGDD mutant was used in this assay to exclude potential effects of RNA replication. Electroporated cells (5 x 10^5^) were seeded onto 6-well plates and incubated at 37°C for 1, 2, 4, or 6 h. The cells were lysed with 350 μl of luciferase lysis buffer per well. The firefly and *Renilla* luciferase activities were measured separately in a Lumat LB 9507 single tube reader (Berthold). *Renilla* counts were used as an internal control for transfection efficiency.

### Viral RNA isolation and quantification

Cells infected by HCV were collected by using TRIzol reagent (Ambion) to purify RNA according to the manufacturer’s protocol. Purified RNAs were quantified by UV absorbance through Nanodrop (Thermo Fisher Scientific), and 500 ng of RNA was used in quantitative RT-PCR.

### Yeast two-hybrid screening

Panbionet performed yeast two-hybrid screening (http://panbionet.com) [59, 60]. NS5A (aa 31–466, genotype 2a), Core (genotype 1a), and NS3 (genotype 2a) genes were amplified by PCR and inserted into a pGBK-T7 bait vector which contains the DNA-binding domain (BD) of GAL4. Screening was performed by using pGBK-T7 containing viral proteins as baits and human liver and thymus cDNA libraries fused with the activation domain (AD) of GAL4 as preys. Yeast strain PBN204 containing two different reporter genes (*URA3* and *ADE2*) was used in colony selection and screening. Transformed yeast cells were applied onto agarose plates with selection media lacking leucine and tryptophan (SD-LW) to select co-transformants of bait and prey vectors. Specific interactions between bait and prey proteins were monitored by yeast cell growth on a selective medium lacking leucine, tryptophan, and adenine (SD-LWA) or on a selective medium lacking leucine, tryptophan, and uracil (SD-LWU). Polypyrimidine tract binding protein (PTB) gene fused with the GAL4 DNA-binding domain (BD-PTB) and PTB gene fused with the GAL4 activation domain (AD-PTB) were used as positive controls of bait and prey vectors, respectively.

### Sample preparation for TEM and ultrastructural analysis

Sample preparation was performed as described previously [38, 49]. Cells were fixed with 2.5% glutaraldehyde (GA), 2% sucrose in 50 mM sodium cacodylate buffer (CaCo), supplemented with 50 mM KCl, 2.6 mM MgCl_2_ and 2.6 mM CaCl_2_ for at least 30 min at room temperature. After five washes with 50 mM CaCo, samples were incubated with 2% osmium tetroxide in 25 mM CaCo for 40 min on ice, washed three times with EM-grade water, and incubated in 0.5% uranyl acetate in water overnight at 4°C. Samples were rinsed three times with water, dehydrated in a graded ethanol series (from 40% to 100%) at room temperature, embedded in Epon 812 (Electron Microscopy Sciences), and polymerized for at least 48 h at 60°C. After polymerization, ultrathin sections of 70 nm were obtained by sectioning with an ultramicrotome Leica EM UC6 (Leica Microsystems) and mounted on a slot grid. Sections were counterstained using 3% uranyl acetate in 70% methanol for 5 min, lead citrate (Reynold’s) for 2 min, and imaged by using a JEOL JEM-1400 (JEOL) operating at 80 kV and equipped with a 4K TemCam F416 (Tietz Video and Image Processing Systems GmBH).

Correlative light and electron microscopy (CLEM) was performed as described previously [61]. In brief, Huh7-Lunet/T7 cells (5 x 10^4^ cells) [62] were seeded onto glass-bottom culture dishes containing gridded coverslips (MatTek Corporation) and then incubated overnight. Cells were transfected with pTM NS3-5B plasmid containing a GFP insertion in NS5A. 24 h after cultivation of transfected cells, differential interference contrast (DIC) and GFP signals were acquired by confocal microscopy with a 10 x objective lens. Cells were fixed with 2.5% GA, 2% sucrose in 50 mM CaCo buffer, supplemented with 50 mM KCl, 2.6 mM MgCl_2_ and 2.6 mM CaCl_2_ for at least 30 min at room temperature. After five washes with CaCo buffer, cells were treated in the same way as for EM sample preparation described above.

### GFP-LC3 puncta formation assay with immunocytochemistry

GFP-LC3 lentivirus was used to generate Huh7 cells stably expressing GFP-LC3 by using Blasticidin S selection method [36]. GFP-LC3 Huh7 cells were inoculated with JC1 or lentiviruses expressing various viral proteins. Two days after infection, the cells were lysed for Western blotting or fixed for immunocytochemistry. The fixed cells were blocked by 1% BSA solution in PBS, and NS5A (Rabbit) or Flag (mouse) antibodies were treated to visualize the protein of interest. Cy5 labeled anti-mouse antibody was used as a secondary antibody, and Hoechst-33258 was used for nucleus staining. Microscopy images were obtained by Custom TCS SP5 II MP confocal microscope (Leica). The images were analyzed by Las AF program, and GFP-LC3 puncta were counted per each cell.

### Quantification of protein bands and statistical analysis

The protein bands of Western blots were quantified by using Image J (V1.52a). Statistical analyses were performed using Prism (GraphPad Software, V8.02). The mean and standard error values in scatterplots are depicted as horizontal bars in the middle and on the top plus bottom, respectively. Statistical significances are depicted by asterisks in figures: (*) for p < 0.05, (**) for p <0.01, (***) for p < 0.001, and (****) for p < 0.0001.

## Supporting information

S1 Fig RACK1 is required for HCV proliferation. (A-B) Effects of RACK1 depletion on HCV proliferation. Huh7.5.1 cells were transfected with 4 different siRNAs against RACK1, and then inoculated with JFH1-ad34-5A-Rluc (0.1 MOI) at 24 h after transfection. Protein levels were monitored by Western blotting, and luciferase activities were measured to monitor HCV proliferation 2 days after infection. Columns and bars represent the mean and SD values of 3 independent experiments. P value was less than 0.0001 (****) by the analysis with ordinary one-way ANOVA multiple comparisons. (C-D) Effects of RACK1 depletion and reconstitution on HCV proliferation. GFP-control and GFP-RACK1 cells were transfected with siRACK1-3 or siRACK1-4, and then inoculated with JFH1-ad34-5A-Rluc (0.1 MOI) at 24 hr after transfection. The siRACK1-3 and siRACK1-4 attack the 3’UTR of endogenous RACK1 mRNAs but not ectopically expressing GFP-RACK1 mRNAs. Protein levels were monitored by Western blotting, and luciferase activities were measured 2 days after infection. Columns and bars represent the mean and SD values of 3 independent experiments. P values were less than 0.01 (**) or 0.0001 (****).
(TIF)

S2 Fig Effect of Rack1 knockdown on HCV IRES-dependent translation. Huh7.5 cells transfected with siRACK1-1 were co-transfected with a reporter replicon RNA (ΔGDD) and a capped *Renilla* transcript (control mRNA) as described in Materials and Methods. The cells were lysed at the indicated time points, and the firefly and *Renilla* luciferase activities reflecting HCV RNA translation and transfection efficiency, respectively, were measured. Arbitrary light units of firefly luciferase were divided by relative values of *Renilla* luciferase activities to normalize variations of transfection efficiencies. Statistical significance was analyzed by t-test. ns stands for non-significant difference.
(TIF)

S3 Fig Determination of the domains responsible for NS5A-RACK1 interaction by yeast two-hybrid assay. (A-C) A yeast strain PBN 204 containing *ADE2* and *URA3* genes under the control of GAL4-binding site was co-transformed with a bait plasmid expressing BD-RACK1 (aa 1–318), BD-RACK1 (aa 120–318), or BD (negative control) and a prey plasmid expressing AD-NS5A (aa 31–249), AD-NS5A (aa 250–466), AD-NS5A (aa 31–213), AD-NS5A (aa 214–338), AD-NS5A (aa 339–466), or AD (negative control). Transformed yeast cells were plated onto selection medium lacking leucine and tryptophan (SD-LW) to select co-transformants (C). Specific interactions between two proteins were monitored by yeast cell growth on (A) a selective medium lacking leucine, tryptophan, and adenine (SD-LWA) or (B) on a selective medium lacking leucine, tryptophan, and uracil (SD-LWU). BD-PTB (polypyrimidine tract binding protein) and AD-PTB served as a positive control for protein-protein interaction.
(TIF)

S4 Fig Domain 1 of NS5A induces autophagy. Representative images of fluorescence microscopy data. Huh7 cells expressing GFP-LC3 (GFP-LC3 Huh7 cells) were used in LC3 puncta formation assays. NS5A variants, NS4B or GST-flag, were expressed by using a pWPI-based lentivirus system. The lentiviruses were inoculated to GFP-LC3 Huh7 cells and cultivated overnight. The cells were further cultivated for 48 h after changing the media. The cells were fixed and analyzed by a fluorescence microscope. Green and red colors in merged images show GFP-LC3 and Flag-tagged NS4B or NS5A variants, respectively. Number of LC3 puncta per cell is presented in (Fig 4B).
(TIF)

S5 Fig RACK1 is required for the autophagy induction by NS5A. Representative images of fluorescence microscopy data. GPF-LC3 Huh7 cells were transfected by RACK1 siRNA. One day post-transfection, lentiviruses expressing either NS5A-WT or NS5A-domain 1 were inoculated to the cells. Cells were fixed 48 h after infection and samples were analyzed by a fluorescence microscope. Green and red colors in merged images show GFP-LC3 and Flag-tagged NS5A variants, respectively. Number of LC3 puncta per cell is presented in (Fig 4D).
(TIF)

S6 Fig RACK1 is necessary to induce autophagy by HCV infection. Representative images of fluorescence microscopy data. GFP-LC3 Huh7 cells were transfected by RACK1 siRNA. One day post-transfection, HCV JC1 was inoculated to the cells. 48 hours after infection, cells were fixed, and samples were analyzed by a fluorescence microscope. Green and red colors in merged images show GFP-LC3 and NS5A, which is visualized by a primary antibody against NS5A, respectively. Number of LC3 puncta per cell is presented in (Fig 4F).
(TIF)

S7 Fig Interactions between vesicle nucleation complex, NS5A and RACK1. (A) Vps15 does not interact with NS5A. Plasmids encoding Flag-tagged Vps15 and GFP-tagged NS5A were co-transfected into HEK293FT cells. 48 hours post-transfection, pulldown experiments were performed with a Flag-resin. The resin-bound proteins were visualized by Western blotting. 2% of Flag-captured proteins were loaded onto the input lanes. WCL, whole cell lysate. The weak band on lane 2 depicted by (*) is likely to be a non-specific one since the plasmid expressing Flag-tagged Vps15 was not transfected in the cells, and no band was detected on the lane visualizing Flag-resin bound proteins (lane 5). Flag antibody was used for Western blotting of Vps15. (B-C) RACK1 does not affect the interaction between NS5A with Beclin1 or Vps34. RACK1 siRNA was transfected into Huh7 cells. One day after the siRNA transfection, plasmids encoding Flag-Beclin1 and GFP-NS5A (B), or Flag-Vps34 and NS5A-HA (C) were co-transfected into the cells. Two days after the DNA transfection, the NS5A-Beclin1 or NS5A-Vps34 interactions were analyzed by Flag-resin precipitation and Western blotting. 4% of Flag-captured proteins were loaded onto the input lanes. WCL, whole cell lysate.
(TIF)

S8 Fig Confirmation of protein-protein interaction between endogenous NS5A and RACK1. (A) Plasmid encoding Flag-tagged NS5A (NS5A-Flag) was transfected into HEK293FT cells. 48 h post-transfection, pulldown experiments were performed with a Flag-resin. The resin-bound proteins were visualized by Western blotting. 3% of cell lysates used in pulldown experiments were loaded onto the input lanes. WCL, whole cell lysate. RACK1 band is indicated by an arrow. (B) Immunoprecipitation experiments were performed with equal amounts of Huh7 cells with (Replicon) or without (Huh7) a HCV replicon using beads conjugated with control- or RACK1-antibodies. The bead-bound proteins were analyzed by Western blotting. 3% of cell lysates used in pulldown experiments were loaded onto the input lanes. NS5A band is indicated by an arrow.
(TIF)

S1 Table Full list of yeast two-hybrid screening. Candidate proteins associate with HCV Core, NS3, and NS5A which were identified by yeast two-hybrid screening. Y2H: yeast two-hybrid.
(XLSX)
