# Supplementary figures and images for: RACK1 mediates rewiring of intracellular networks induced by hepatitis C virus infection
Source: PLoS Pathog. 2019 Sep 16;15(9):e1008021. doi: 10.1371/journal.ppat.1008021 (PMC6762199; doi:10.1371/journal.ppat.1008021)

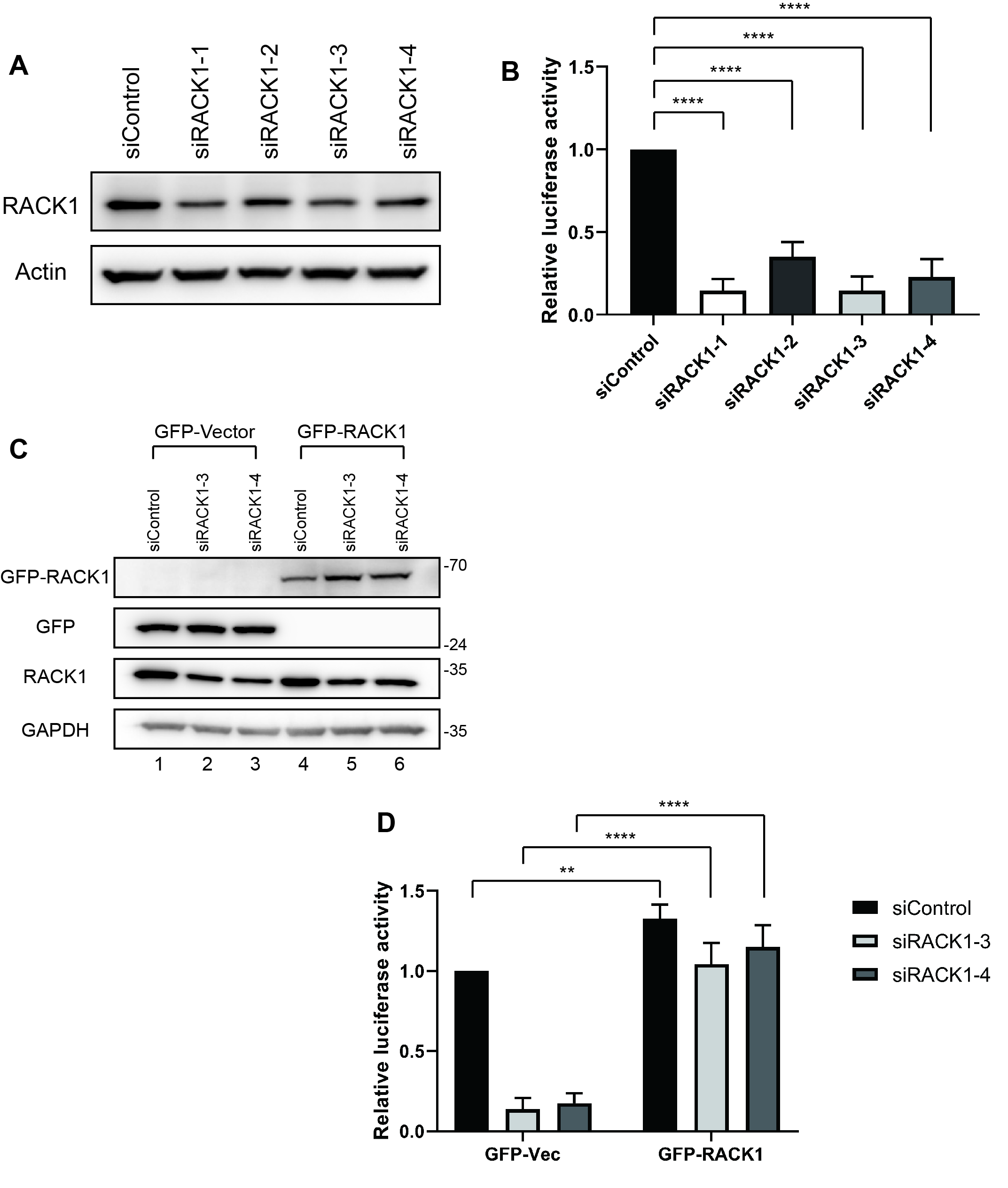

Supplement: S1 Fig — (A-B) Effects of RACK1 depletion on HCV proliferation. Huh7.5.1 cells were transfected with 4 different siRNAs against RACK1, and then inoculated with JFH1-ad34-5A-Rluc (0.1 MOI) at 24 h after transfection. Protein levels were monitored by Western blotting, and luciferase activities were measured to monitor HCV proliferation 2 days after infection. Columns and bars represent the mean and SD values of 3 independent experiments. P value was less than 0.0001 (****) by the analysis with ordinary one-way ANOVA multiple comparisons. (C-D) Effects of RACK1 depletion and reconstitution on HCV proliferation. GFP-control and GFP-RACK1 cells were transfected with siRACK1-3 or siRACK1-4, and then inoculated with JFH1-ad34-5A-Rluc (0.1 MOI) at 24 hr after transfection. The siRACK1-3 and siRACK1-4 attack the 3’UTR of endogenous RACK1 mRNAs but not ectopically expressing GFP-RACK1 mRNAs. Protein levels were monitored by Western blotting, and luciferase activities were measured 2 days after infection. Columns and bars represent the mean and SD values of 3 independent experiments. P values were less than 0.01 (**) or 0.0001 (****). (TIF) [file ppat.1008021.s001.tif]

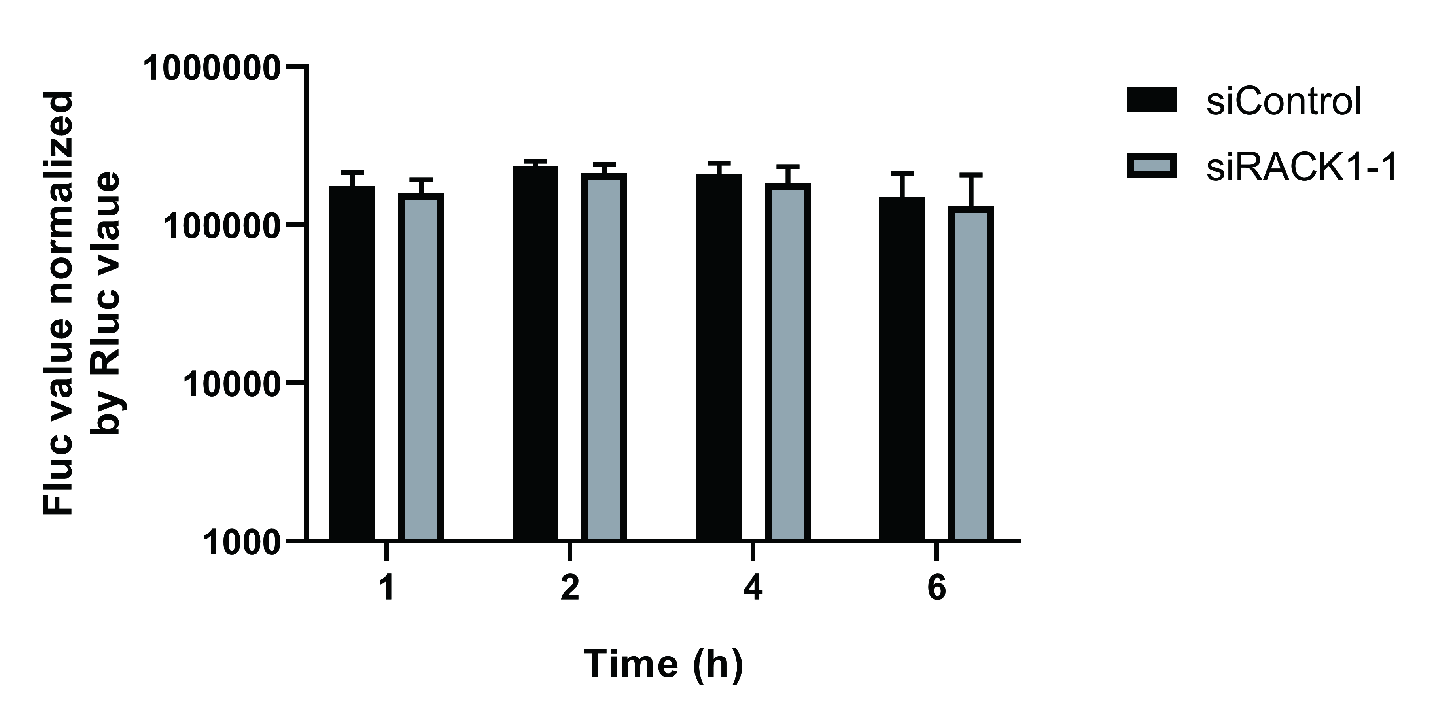

Supplement: S2 Fig — Huh7.5 cells transfected with siRACK1-1 were co-transfected with a reporter replicon RNA (ΔGDD) and a capped Renilla transcript (control mRNA) as described in Materials and Methods. The cells were lysed at the indicated time points, and the firefly and Renilla luciferase activities reflecting HCV RNA translation and transfection efficiency, respectively, were measured. Arbitrary light units of firefly luciferase were divided by relative values of Renilla luciferase activities to normalize variations of transfection efficiencies. Statistical significance was analyzed by t-test. ns stands for non-significant difference. (TIF) [file ppat.1008021.s002.tif]

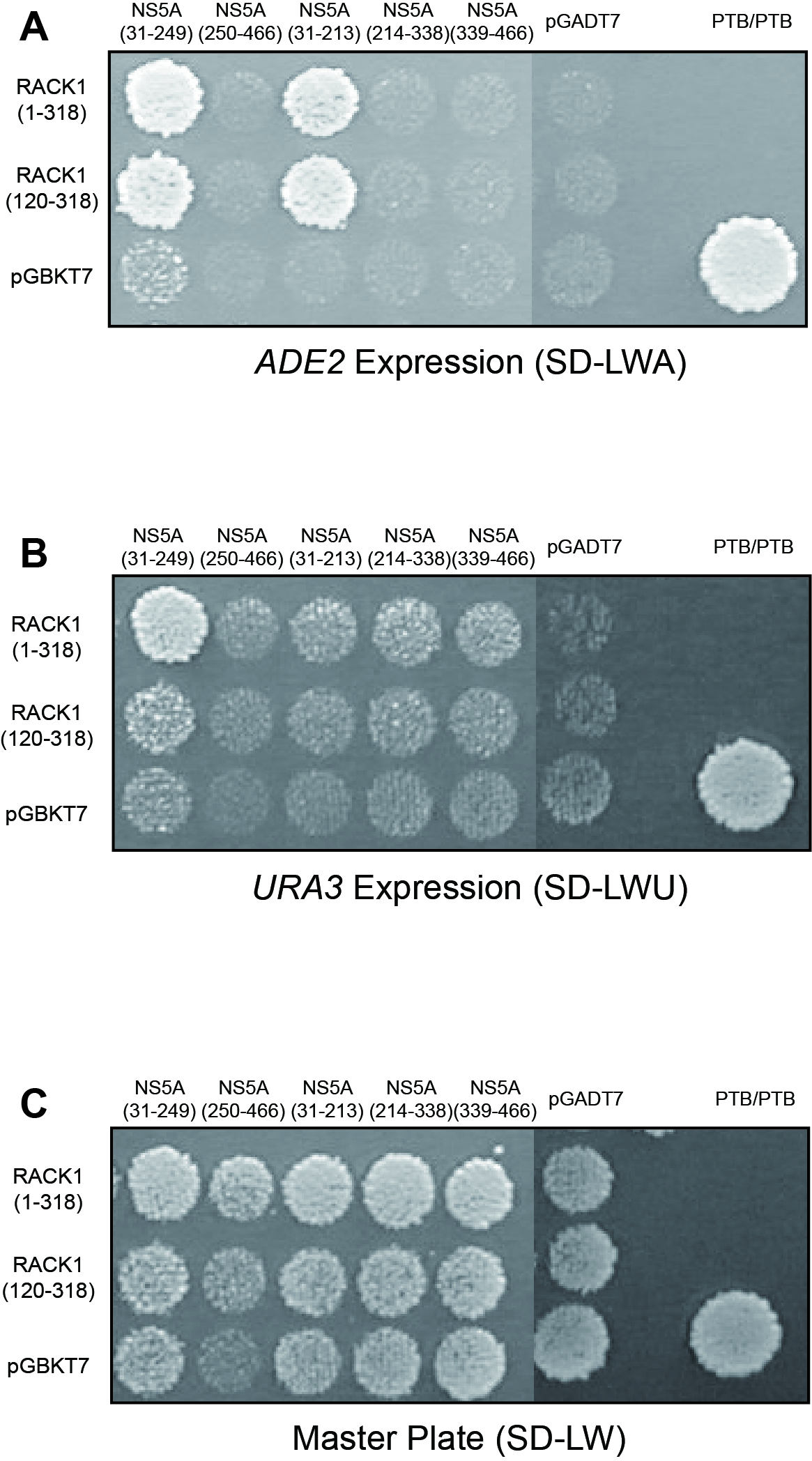

Supplement: S3 Fig — (A-C) A yeast strain PBN 204 containing ADE2 and URA3 genes under the control of GAL4-binding site was co-transformed with a bait plasmid expressing BD-RACK1 (aa 1–318), BD-RACK1 (aa 120–318), or BD (negative control) and a prey plasmid expressing AD-NS5A (aa 31–249), AD-NS5A (aa 250–466), AD-NS5A (aa 31–213), AD-NS5A (aa 214–338), AD-NS5A (aa 339–466), or AD (negative control). Transformed yeast cells were plated onto selection medium lacking leucine and tryptophan (SD-LW) to select co-transformants (C). Specific interactions between two proteins were monitored by yeast cell growth on (A) a selective medium lacking leucine, tryptophan, and adenine (SD-LWA) or (B) on a selective medium lacking leucine, tryptophan, and uracil (SD-LWU). BD-PTB (polypyrimidine tract binding protein) and AD-PTB served as a positive control for protein-protein interaction. (TIF) [file ppat.1008021.s003.tif]

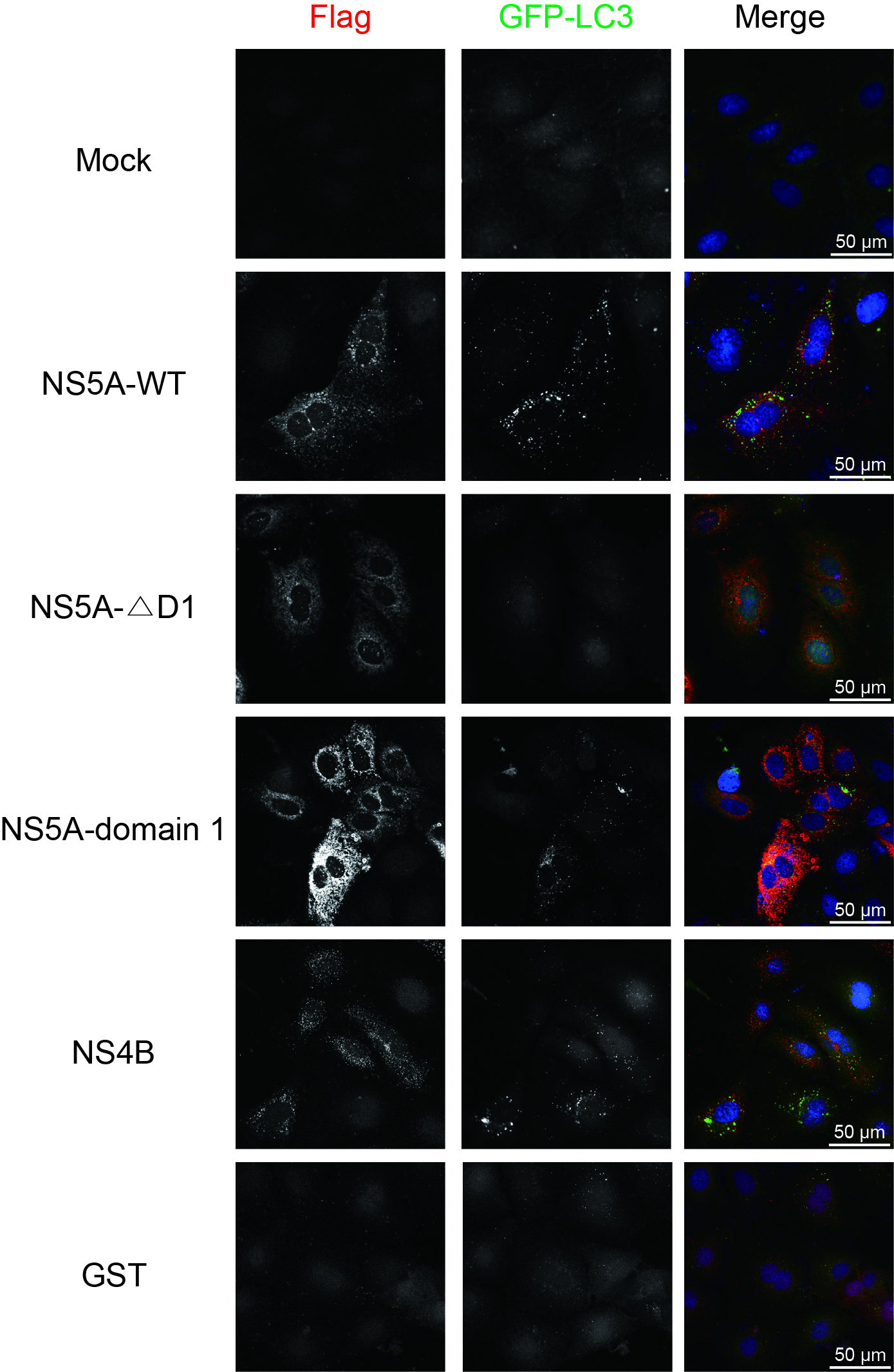

Supplement: S4 Fig — Representative images of fluorescence microscopy data. Huh7 cells expressing GFP-LC3 (GFP-LC3 Huh7 cells) were used in LC3 puncta formation assays. NS5A variants, NS4B or GST-flag, were expressed by using a pWPI-based lentivirus system. The lentiviruses were inoculated to GFP-LC3 Huh7 cells and cultivated overnight. The cells were further cultivated for 48 h after changing the media. The cells were fixed and analyzed by a fluorescence microscope. Green and red colors in merged images show GFP-LC3 and Flag-tagged NS4B or NS5A variants, respectively. Number of LC3 puncta per cell is presented in (Fig 4B). (TIF) [file ppat.1008021.s004.tif]

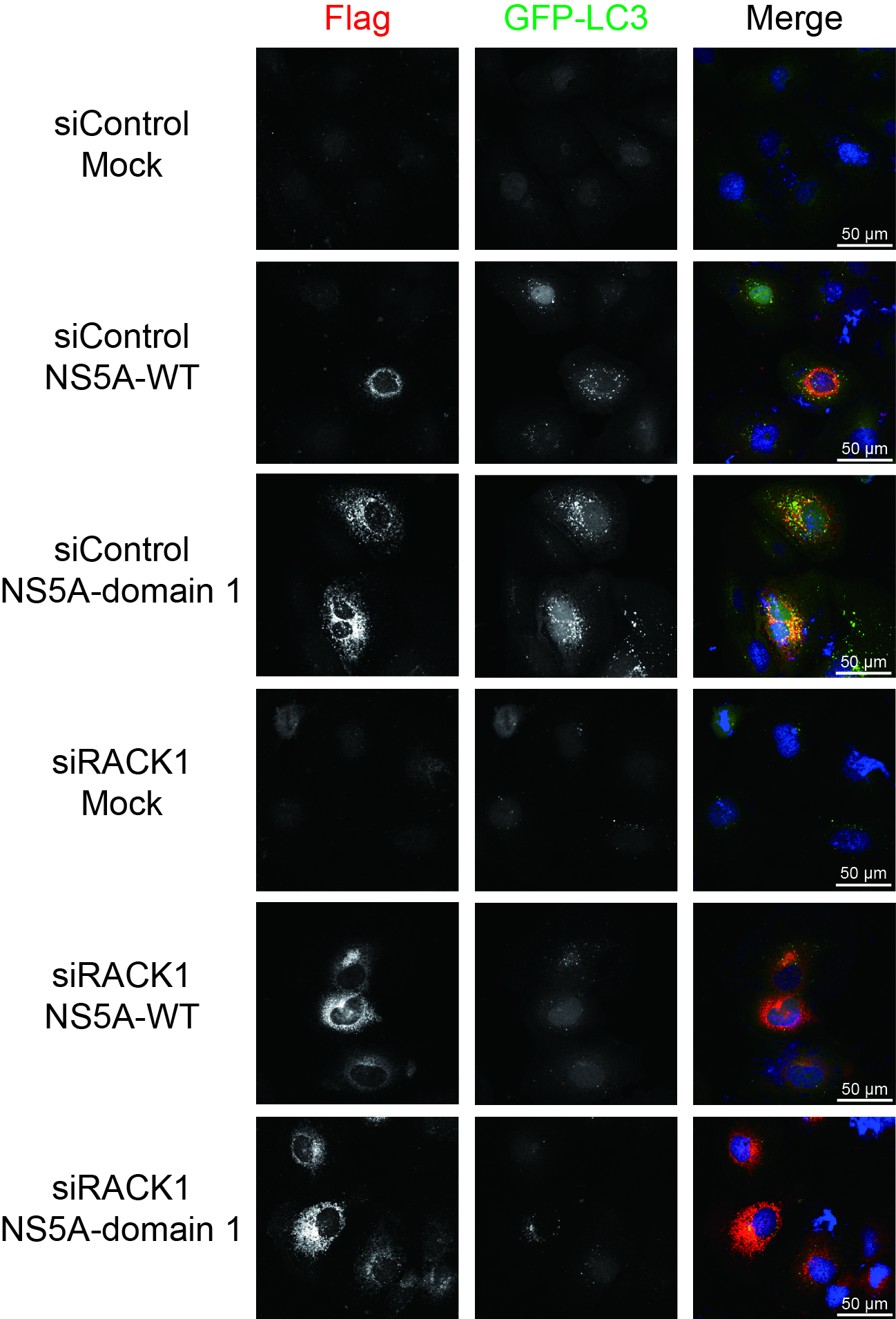

Supplement: S5 Fig — Representative images of fluorescence microscopy data. GPF-LC3 Huh7 cells were transfected by RACK1 siRNA. One day post-transfection, lentiviruses expressing either NS5A-WT or NS5A-domain 1 were inoculated to the cells. Cells were fixed 48 h after infection and samples were analyzed by a fluorescence microscope. Green and red colors in merged images show GFP-LC3 and Flag-tagged NS5A variants, respectively. Number of LC3 puncta per cell is presented in (Fig 4D). (TIF) [file ppat.1008021.s005.tif]

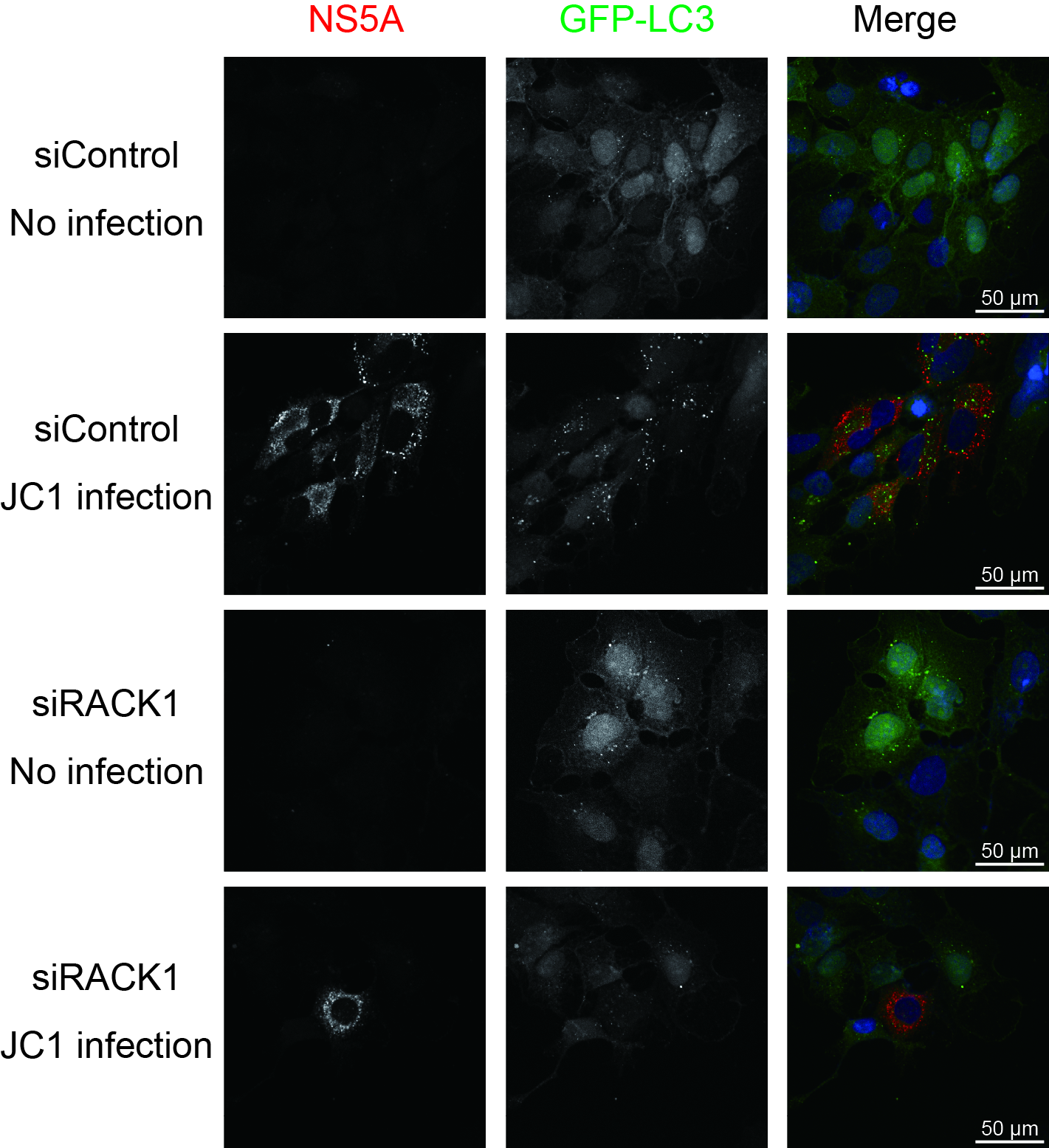

Supplement: S6 Fig — Representative images of fluorescence microscopy data. GFP-LC3 Huh7 cells were transfected by RACK1 siRNA. One day post-transfection, HCV JC1 was inoculated to the cells. 48 hours after infection, cells were fixed, and samples were analyzed by a fluorescence microscope. Green and red colors in merged images show GFP-LC3 and NS5A, which is visualized by a primary antibody against NS5A, respectively. Number of LC3 puncta per cell is presented in (Fig 4F). (TIF) [file ppat.1008021.s006.tif]

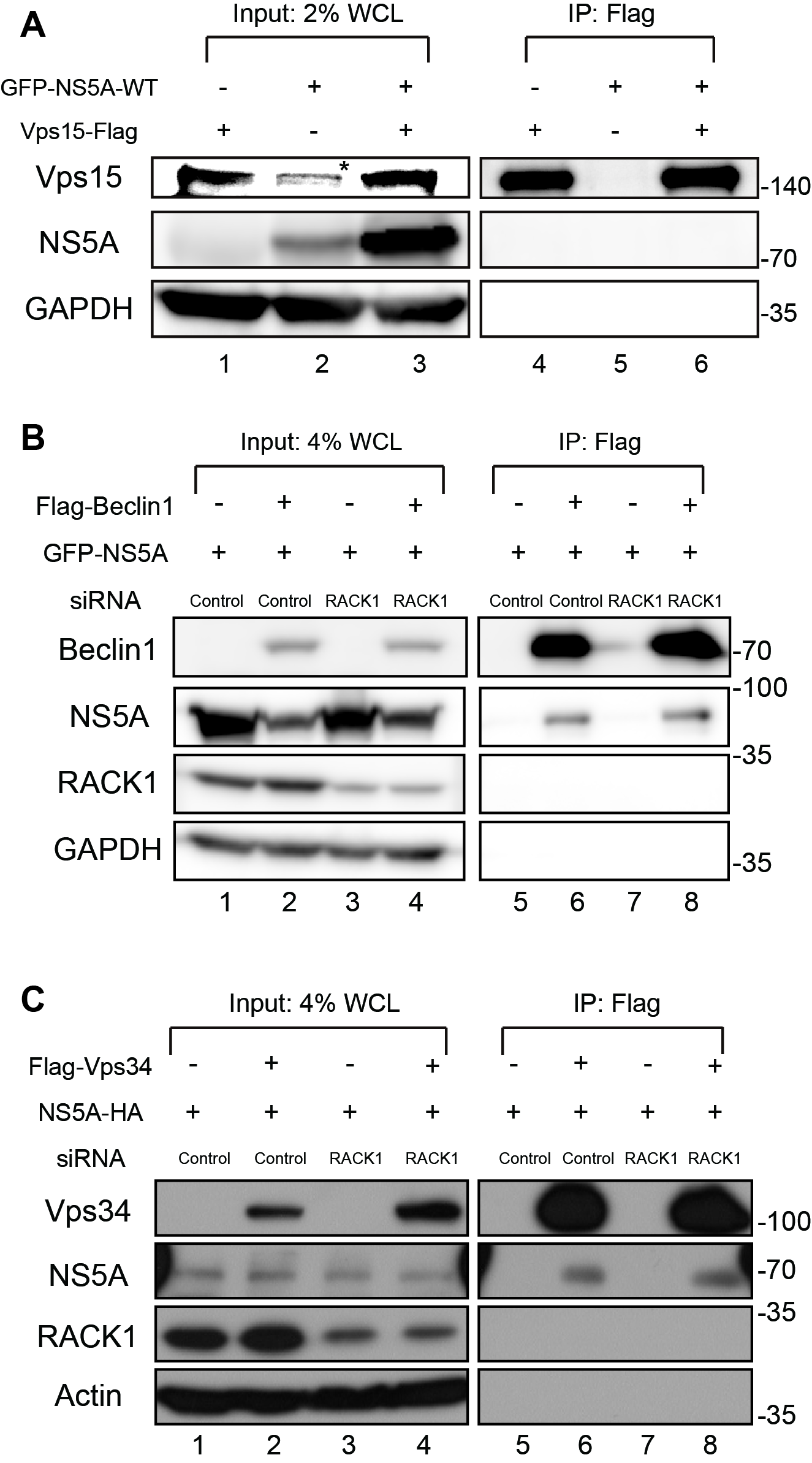

Supplement: S7 Fig — (A) Vps15 does not interact with NS5A. Plasmids encoding Flag-tagged Vps15 and GFP-tagged NS5A were co-transfected into HEK293FT cells. 48 hours post-transfection, pulldown experiments were performed with a Flag-resin. The resin-bound proteins were visualized by Western blotting. 2% of Flag-captured proteins were loaded onto the input lanes. WCL, whole cell lysate. The weak band on lane 2 depicted by (*) is likely to be a non-specific one since the plasmid expressing Flag-tagged Vps15 was not transfected in the cells, and no band was detected on the lane visualizing Flag-resin bound proteins (lane 5). Flag antibody was used for Western blotting of Vps15. (B-C) RACK1 does not affect the interaction between NS5A with Beclin1 or Vps34. RACK1 siRNA was transfected into Huh7 cells. One day after the siRNA transfection, plasmids encoding Flag-Beclin1 and GFP-NS5A (B), or Flag-Vps34 and NS5A-HA (C) were co-transfected into the cells. Two days after the DNA transfection, the NS5A-Beclin1 or NS5A-Vps34 interactions were analyzed by Flag-resin precipitation and Western blotting. 4% of Flag-captured proteins were loaded onto the input lanes. WCL, whole cell lysate. (TIF) [file ppat.1008021.s007.tif]

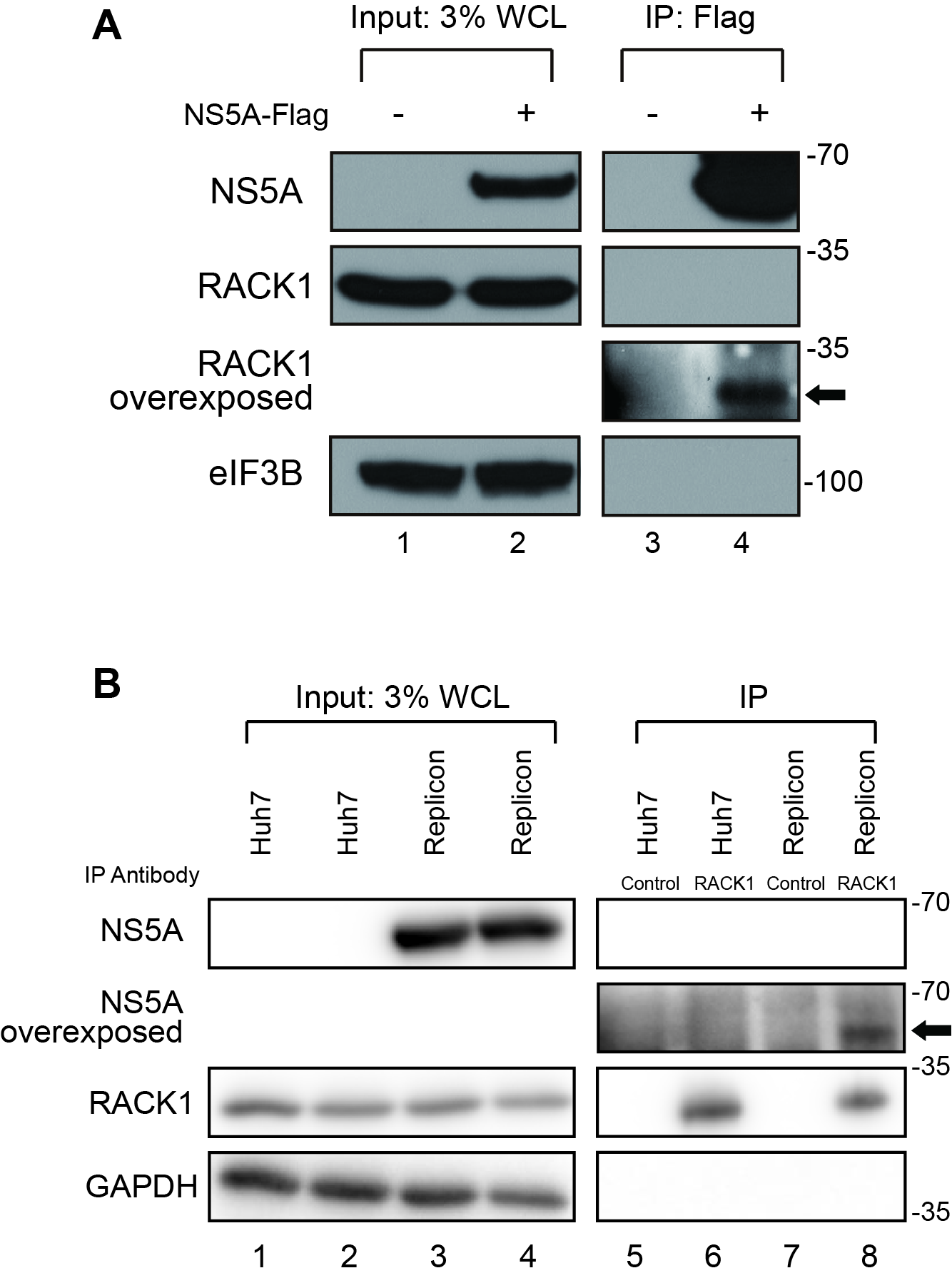

Supplement: S8 Fig — (A) Plasmid encoding Flag-tagged NS5A (NS5A-Flag) was transfected into HEK293FT cells. 48 h post-transfection, pulldown experiments were performed with a Flag-resin. The resin-bound proteins were visualized by Western blotting. 3% of cell lysates used in pulldown experiments were loaded onto the input lanes. WCL, whole cell lysate. RACK1 band is indicated by an arrow. (B) Immunoprecipitation experiments were performed with equal amounts of Huh7 cells with (Replicon) or without (Huh7) a HCV replicon using beads conjugated with control- or RACK1-antibodies. The bead-bound proteins were analyzed by Western blotting. 3% of cell lysates used in pulldown experiments were loaded onto the input lanes. NS5A band is indicated by an arrow. (TIF) [file ppat.1008021.s008.tif]
